# The potential crosstalk between tumor and plasma cells and its association with clinical outcome and immunotherapy response in bladder cancer

**DOI:** 10.1186/s12967-023-04151-1

**Published:** 2023-05-03

**Authors:** Fei Long, Wei Wang, Shuo Li, Bicheng Wang, Xin Hu, Jun Wang, Yaqi Xu, Min Liu, Junting Zhou, Huaqi Si, Xiaodan Xi, Xiang-yu Meng, Chunhui Yuan, Fubing Wang

**Affiliations:** 1 Department of Laboratory Medicine, Zhongnan Hospital of Wuhan University, Wuhan, China; 2 Center for Single-Cell Omics and Tumor Liquid Biopsy, Zhongnan Hospital of Wuhan University, Wuhan, China; 3 Department of Laboratory Medicine, Wuhan Children’s Hospital (Wuhan Maternal and Child Healthcare Hospital), Tongji Medical College, Huazhong University of Science & Technology, Wuhan, China; 4 Wuhan Research Center for Infectious Diseases and Cancer, Chinese Academy of Medical Sciences, Wuhan, China; 5 Department of Pathology, Zhongnan Hospital of Wuhan University, Wuhan, China; 6 College of Basic Medical Sciences, Medical School, Hubei Minzu University, Enshi, China

**Keywords:** Cell crosstalk, Plasma cell, Bladder cancer, Immunotherapy, Single-cell analysis

## Abstract

**Background:**

Although immunotherapy is effective in improving the clinical outcomes of patients with bladder cancer (BC), it is only effective in a small percentage of patients. Intercellular crosstalk in the tumor microenvironment strongly influences patient response to immunotherapy, while the crosstalk patterns of plasma cells (PCs) as endogenous antibody-producing cells remain unknown. Here, we aimed to explore the heterogeneity of PCs and their potential crosstalk patterns with BC tumor cells.

**Methods:**

Crosstalk patterns between PCs and tumor cells were revealed by performing integrated bulk and single-cell RNA sequencing (RNA-seq) and spatial transcriptome data analysis. A risk model was constructed based on ligand/receptor to quantify crosstalk patterns by stepwise regression Cox analysis.

**Results:**

Based on cell infiltration scores inferred from bulk RNA-seq data (n = 728), we found that high infiltration of PCs was associated with better overall survival (OS) and response to immunotherapy in BC. Further single-cell transcriptome analysis (n = 8; 41,894 filtered cells) identified two dominant types of PCs, IgG1 and IgA1 PCs. Signal transduction from tumor cells of specific states (stress-like and hypoxia-like tumor cells) to PCs, for example, via the LAMB3/CD44 and ANGPTL4/SDC1 ligand/receptor pairs, was validated by spatial transcriptome analysis and associated with poorer OS as well as nonresponse to immunotherapy. More importantly, a ligand/receptor pair-based risk model was constructed and showed excellent performance in predicting patient survival and immunotherapy response.

**Conclusions:**

PCs are an important component of the tumor microenvironment, and their crosstalk with tumor cells influences clinical outcomes and response to immunotherapies in BC patients.

**Graphical Abstract:**

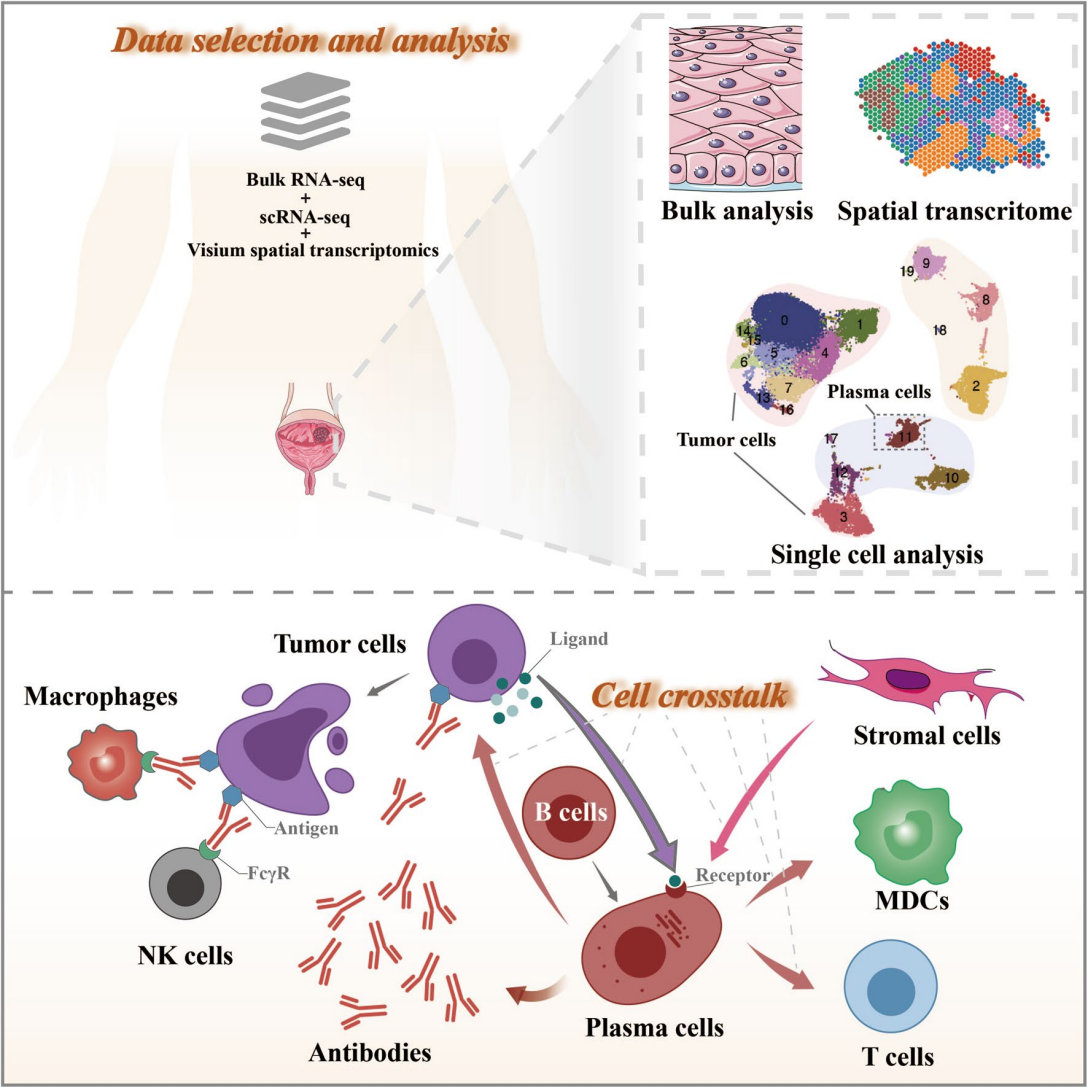

**Supplementary Information:**

The online version contains supplementary material available at 10.1186/s12967-023-04151-1.

## Background

Bladder cancers (BCs) (> 90%) mainly originate from the deterioration of epithelial cells; there are more than 570,000 new cases of BC in 2020, with BC ranking sixth among all cancers in males. Because BC exhibits inherently aggressive features and is prone to recurrence, patients usually require multimodal and invasive treatments, such as repeated endoscopic resection or transurethral resection of bladder tumor (TURBT), as well as first-line platinum-based chemotherapy, but the 5-year relapse-free survival is still less than 43% [1].

In the past decade, the treatment landscape of BC has changed dramatically because immune checkpoint inhibitors (ICIs) have a better safety profile and induce more durable responses than platinum-based chemotherapy [2, 3]. The National Comprehensive Cancer Network guidelines recommend ICIs, such as the PD-1 blocker pembrolizumab and the PD-L1 blocker atezolizumab, as first-/second-line treatment options for patients with metastatic BC who are ineligible for platinum-based chemotherapy [4, 5]. Unfortunately, the therapeutic effect of ICIs in BC is limited, with an objective response rate of less than 30% [6]. Therefore, an in-depth analysis of the potential factors affecting the efficacy of ICIs is important to further improve the clinical management and prognosis of patients.

Responses to ICI therapies can be influenced by multiple factors, such as mutational, transcriptomic, and epigenetic alterations, as well as a suppressive tumor immune microenvironment (TIME) [7, 8]. Nevertheless, ICIs boost the activation of the immune system to attack cancer cells, and thus, exploration of cellular communication between tumor and immune cells in the TIME is pivotal to maximizing the clinical benefit of ICIs [9, 10]. Indeed, a specific cluster of cancer cells in BC can influence patient response to chemotherapy or immunotherapy by communicating with cancer-associated fibroblasts (CAFs) and CD8^+^ T cells [11]. Tumor-associated tertiary lymphoid structures (TLSs) have been recently discovered and are associated with better patient survival and immune response in BC [12, 13]. In contrast to previous knowledge, TLSs contain not only a high abundance of T cells but also substantial infiltration of ICI-responsive B cells [14], which further highlights the complexity of cellular communication in the TIME.

Plasma cells (PCs) are a pivotal group of B cells in TLSs and can produce large amounts of cytokines and IgG or IgA antibodies targeting tumor-associated antigens in situ. Known roles include driving antibody-dependent cell-mediated cytotoxicity (ADCC), promoting phagocytosis, and enhancing antigen presentation by dendritic cells [15, 16]. Importantly, high frequencies of IgG-producing PCs are associated with therapeutic responses to ICIs in various cancers, such as renal cell carcinoma (RCC) [17], non-small cell lung cancer (NSCLC) [18] and BC [19]. To date, tumor-associated macrophages, fibroblasts and neutrophils have been reported to interact with B cells and mediate their differentiation into PCs [20, 21]. However, it is still not clear whether PCs directly interact with tumor cells and what role tumor cell-PC communication plays in BC.

**Fig. 1:**
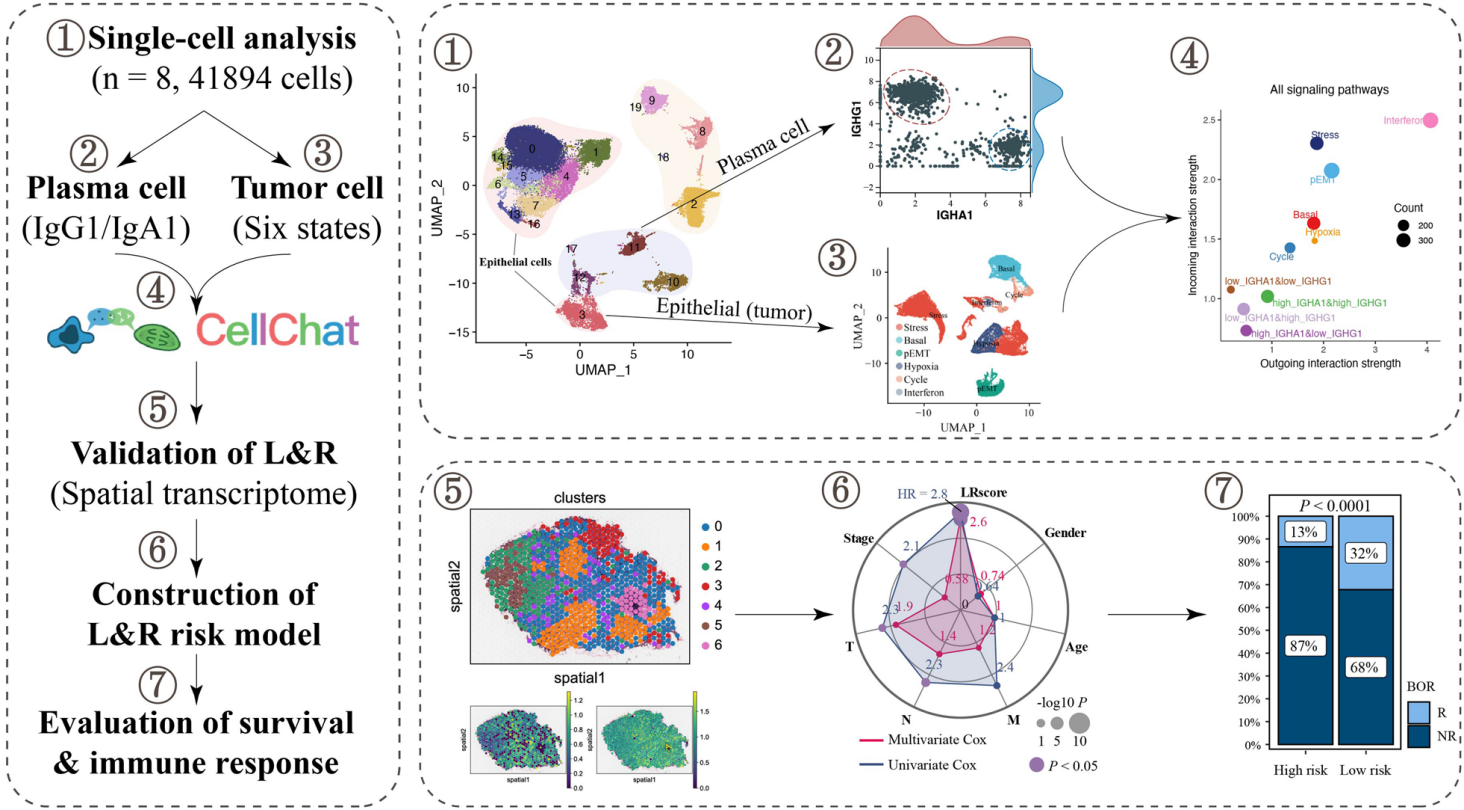
The workflow of this study

In this study, a thorough investigation was conducted to unveil how PCs communicate with tumor cells and the subsequent effects on patients’ response to ICIs and clinical outcomes in BCs. We first performed an scRNA-seq survey of 41,894 cells from eight patients and explored the communication patterns between tumor cells and PCs (Fig. 1). Then, we further identified key ligand/receptor pairs by spatial transcriptome expression analysis to elucidate the crosstalk between tumor cells of specific states and PCs. Finally, we quantified the overall crosstalk between tumor cells and PCs to assess its impact on patient survival and response to ICB therapies. This multidimensional integrated analysis provides unprecedented insights into the cellular heterogeneity and crosstalk patterns of PCs in patients with BC.

## Methods

### Data retrieval and preprocessing

The processed log_2_(count + 1) bulk RNA-seq data (n = 430), MuTect2-processed somatic mutation data and corresponding clinical information of bladder urothelial carcinoma (BLCA) patients from The Cancer Genome Atlas (TCGA) were obtained using the UCSC Xena browser (https://xenabrowser.net/datapages/). RNA-seq data of the anti-PD-L1 therapy cohort (IMvigor210, n = 298) and sample annotation information, including binary best overall response (BOR), tumor mutation burden (TMB), tumor neoantigen burden (TNB), and survival information, were obtained using the IMvigor210CoreBiologies R package [22]. IMvigor210 was a single arm phase II study in patients with metastatic urothelial carcinoma (NCT02108652, NCT02951767) treated with atezolizumab (1200 mg 3 weekly). The primary endpoint was a partial response rate (PR) of more than 10% (RECIST v1.1). Raw scRNA-seq FASTQ data from two low-grade and six high-grade BC samples provided by the Union Hospital of Tongji Medical College, Wuhan, China (PRJNA662018) [23], were obtained from the Sequence Read Archive (SRA). The FASTQ data were filtered and read aligned using CellRanger (v.3.0.1, 10 × Genomics) [24], and feature barcode unique molecular identifier (UMI) matrices were generated based on the human reference genome GRCh38. Visium spatial transcriptomics data (GSE171351) [11] including h5ad files and raw were images downloaded from the Gene Expression Omnibus (GEO) and processed and analyzed using the Python package Scanpy (v.1.9.1) [25]. Briefly, spatial coordinates with total counts greater than 20,000, number of expressed genes greater than 6,000, and mitochondrial genes accounting for greater than 10% were filtered. The "normalize_total" function was used to normalize the counts and spatial information data, and the "highly_variable_genes" function was used to extract the top 2000 highly variable genes, followed by dimension reduction and clustering through principal component analysis (PCA), uniform manifold approximation and projection (UMAP) and Leiden algorithm analysis.

### Inference of immune cell infiltration based on bulk RNA-seq data

To assess the infiltration of immune cells in patients with BC, cell infiltration analysis was performed on bulk RNA-seq data using the R package IOBR [26] with the xCell method [27]. xCell is a method for scoring the abundances of 64 cell types based on single-sample gene set enrichment analysis (ssGSEA). Adaptive and innate immune cells were selected for further analysis. The list of marker genes in cells used in the calculations was downloaded from the study of Aran et al. [27]. Next, univariate Cox regression analysis was performed based on cell scores and patient survival data via the R package survival to predict the effect of cell infiltration scores on overall survival (OS), and Cox *P* < 0.05 was considered significantly associated with patient survival.

### ScRNA-seq data analysis

Cells were first filtered using the R package Seurat [28] using the following criteria: the number of expressed genes was lower than 300 or larger than 6000, and cells with more than 10% of UMIs mapped to mitochondrial genes were excluded. Only genes that were expressed in at least five cells were retained. After that, the data were normalized, and the top 2000 highly variable genes were detected by the "FindVariableFeatures" function. Next, the dimension of the scRNA-seq data was reduced by PCA based on 2000 genes, and 40 principal components were selected for subsequent analysis. Moreover, to remove the batch effects between samples, soft k-means clustering was performed using the R package harmony [29]. Finally, the "FindClusters" function was adopted for cell clustering, and the resolution was set to 0.5. To determine the cell types, we annotated the cells based on the CellMarker database [30] and corresponding literature.

### Analysis of subclusters of PCs

Based on the cell annotation, PCs were extracted by the "subset" function, and dimension reduction and clustering were performed according to the same procedure as above, with resolution set to 0.4. To further identify IgG1- and IgA1-dominant cell subclusters, we classified cells according to the median expression of *IGHG1* and *IGHA1*. Trajectory analysis was performed using Monocle 2 [31] to understand the transformation relationships between different types of PCs. Finally, to reveal the effects of IgG1 and IgA1 PCs on patient survival, an algorithm based on cosine similarity was first adopted using the R package COSG [32] to obtain the top 100 marker genes (Additional file 2: Table S1) of IgG1 or IgA1 PCs. The TCGA-BLCA samples were then grouped by the BC subtypes reported by Kamoun et al*.* [33], and ssGSEA was performed to estimate the cell abundance score for each patient using the R package GSVA based on the top 10 marker genes from the top 100 genes. Samples were grouped using the best cutoff or median based on cell abundance score, and survival analysis was performed using the R package survminer.

### Epithelial (tumor) cell state analysis

To identify malignant cells with clonal large-scale chromosomal copy number variations (CNVs), the CNVs of each cell were inferred using the R package infercnv [34]. We calculated the CNV score for each cell using the method of Peng et al. [35]. To determine the cell state, a nonnegative matrix factorization (NMF) algorithm was first employed for dimension reduction of all tumor cells, and subsequently, cells were scored for state using the R package AUCell [36] based on the gene sets of 12 cell states reported by Barkley et al. [8]. Finally, the R package COSG was used to screen all marker genes of cell state clusters (top 100) (Additional file 3: Table S2), and Gene Ontology (GO) [37], Kyoto Encyclopedia of Genes and Genomes (KEGG) [38], WikiPathways (WP) [39] and Reactome (REAC) [40] enrichment analyses were performed using g:Profiler [41]. The *P* value was adjusted by the g:SCS method.

### Cell communication analysis

To explore the crosstalk pattern between cells, we employed the R package CellChat [42]. Briefly, we followed the official workflow and created the CellChat object based on a normalized count matrix. Subsequently, the "identifyOverExpressedGenes" and "identifyOverExpressedInteractions" functions were used for data processing with a standard parameter set. The potential ligand/receptor (L/R) interactions among all cells, especially the interactions between PCs and tumor cells, were calculated and analyzed based on the functions "computeCommunProb", "computeCommunProbPathway", and "aggregateNet" using standard parameters.

### Risk model construction based on L/R pairs

To quantify the impact of communication between PCs and tumor cells on patient survival, we performed stepwise Cox regression of all L/R pairs (Additional file 4: Table S3) between PCs and tumor cells identified by CellChat using the R package My.stepwise, and the best multivariate Cox regression model based on TCGA-BLCA data was selected based on the Akaike information criterion. Next, we extracted the model coefficient (coef) of each L/R pair and calculated the risk score using the following formula:

$$\mathrm{LRscore }= \sum_{i}{Expression(L or R)}_{i}*{coef}_{i}$$

The median risk score or optimal cutoff values were used to divide the patients into low- and high-risk groups. OS, disease-specific survival (DSS), disease-free survival (DFS), and progression-free survival (PFS) were analyzed using the Kaplan‒Meier method. The R package timeROC was used to assess the predictive power of the LRscore and clinical indicators of patient survival over time. The R package pROC was used to perform ROC analysis for the LRscore and selected anti-PD-L1 treatment-related indicators to compare their ability to discriminate between responding (R) and nonresponding (NR) patients. Multi-indicator ROC assessment was performed using binary logistic regression with linear fitting of multiple indicators based on SPSS (v.25.0).

### Correlation analysis of the LRscore

To assess the association between the LRscore and cancer progression, we first calculated the cancer hallmark score for each sample based on the hallmark gene sets from MSigDB (http://www.gsea-msigdb.org/) using gene set variation analysis (GSVA) and subsequently calculated the correlation coefficient between the LRscore and cancer hallmark score using Spearman correlation analysis. The immune cell infiltration, immune, stromal, and ESTIMATE scores [43], tumor immune dysfunction and exclusion (TIDE) score [44], immunophenoscore (IPS) [45] and associated indicators were calculated using the R package IOBR [26], and their correlations with the LRscore were also evaluated by Spearman correlation analysis.

### Risk group comparison and mutation analysis

The classic subtype of BC reported by Robertson et al*.* [46] and the newest subtype reported by Kamoun et al*.* [33] were compared with our LRscore risk groups using the R package ggalluvial. The mutation profiles of the two risk group samples were analyzed using the R package maftools [47], and the significant differences between the two groups were evaluated using the chi-square test. To assess the response to targeted drugs in different risk groups, model training was performed using the R package oncoPredict [48] based on targeted drug-treated cell expression data from the GDSC database [49] to infer IC50 values for patients in the low- and high-risk groups. A low IC50 value generally indicates that a patient is more sensitive to a drug.

### Statistical analysis

Statistical analysis and visualization were performed using R (v.4.1.1), Python (v.3.10), and Sangerbox [50]. The Wilcoxon test was used to compare two groups because the data were not normally distributed. The Kruskal–Wallis test was used to compare three or more groups. The *P* value was adjusted using the Benjamini and Hochberg method, if necessary. The detailed tools, methods, and thresholds for each analysis are described in the Materials and Methods section or the figure legends.

## Results

### PCs were associated with patient survival and response to immunotherapy

**Fig. 2:**
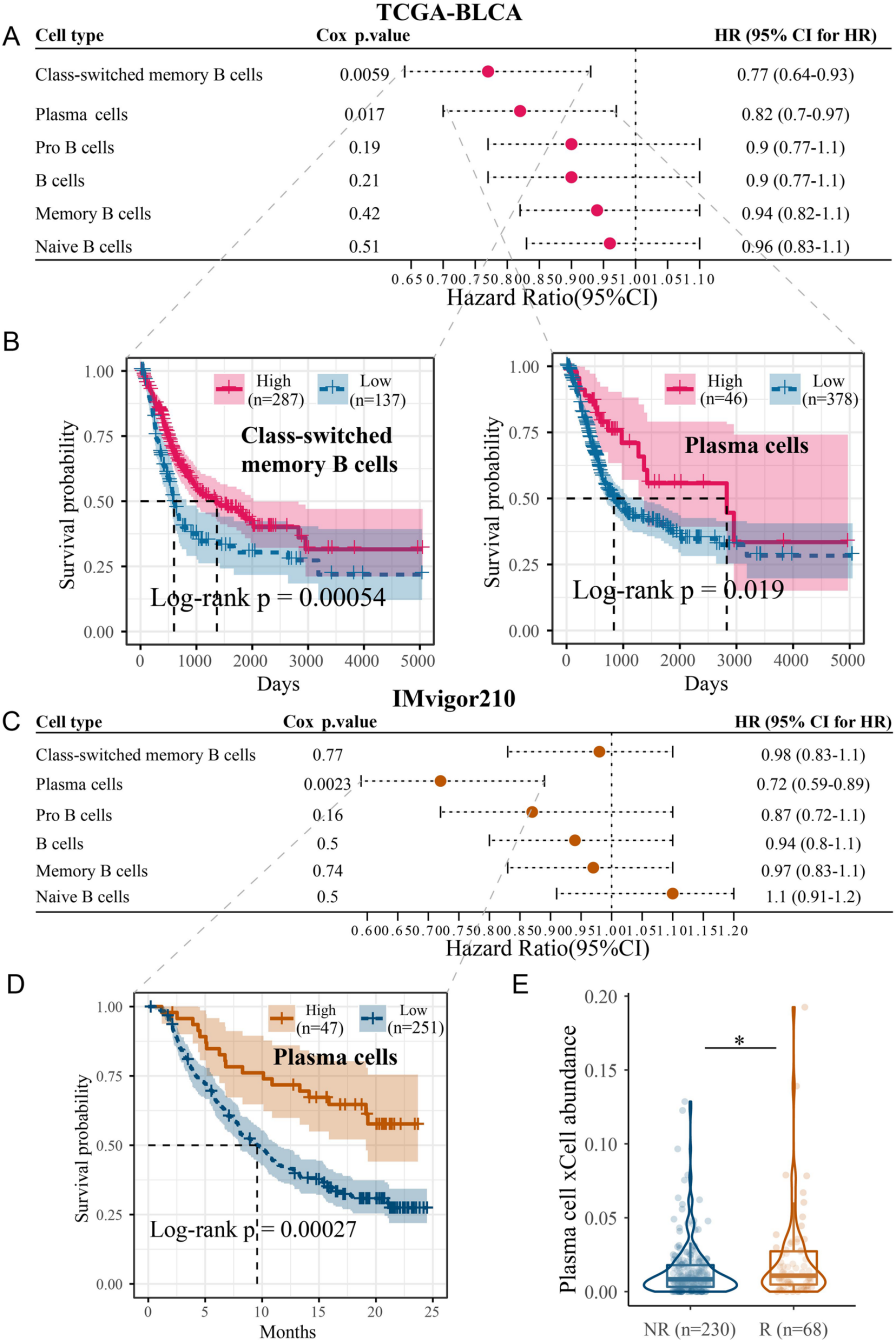
Plasma cell infiltration levels inferred from bulk RNA-seq data correlate with survival and immunotherapy response. **A** Association between inferred B-cell subtype infiltration levels and patient overall survival in the TCGA-BLCA cohort (Cox regression analysis). **B** Kaplan–Meier curves for TCGA-BLCA patients. The *P* value was calculated with the log-rank test. **C** Association between inferred B-cell subtype infiltration levels and patient overall survival in the anti-PD-L1 IMvigor210 cohort (Cox regression analysis). **D** Kaplan–Meier curves for IMvigor210 patients. The *P* value was calculated with the log-rank test. **E** Box and violin composite plots showing a higher plasma cell infiltration level in the response (R) group than in the nonresponse (NR) group. Wilcoxon test; *P < 0.05

Inferential analysis of cellular infiltration (see Materials and Methods 2.2) based on TCGA-BLCA bulk RNA-seq data revealed that 11 immune cell types had a significant association with OS in the univariate Cox regression model (Additional file 1: Figure S1A), with high infiltration of CD8^+^ T_em_ cells, CD8^+^ T cells, NK cells, Th1 cells, and CD8^+^ T_cm_ cells associated with better OS (Cox *P* < 0.05), whereas high infiltration of four cell types (mast cells, neutrophils, M2 macrophages, and endothelial cells) was associated with worse OS (Cox *P* < 0.05). Among all B-cell types, only class-switched memory B cells (Cox *P* = 0.0059; log-rank *P* = 0.00054; *HR* = 0.77) and PCs (Cox *P* = 0.017; log-rank *P* = 0.019; *HR* = 0.82) were significantly associated with better OS (Fig. 2A and B). In the anti-PD-L1-treated IMvigor210 cohort, high infiltration of γδT (Tgd) cells, Th2 cells, and basophils was associated with better OS (Cox *P* < 0.05) (Additional file 1: Figure S1B). Among all B cells, only PCs were associated with better OS (Cox *P* = 0.0023; log-rank *P* = 0.0003; *HR* = 0.72) (Fig. 2C and D). Additionally, immunotherapy-responsive patients had a relatively higher PC abundance (Wilcoxon test, *P* < 0.05) (Fig. 2E). Overall, these bulk sequencing data-based cellular inference results suggest that high overall PC infiltration in BC patients predicts better OS and response to immunotherapy.

### IgG1 and IgA1 were the two major subtypes of PCs in BC

**Fig. 3:**
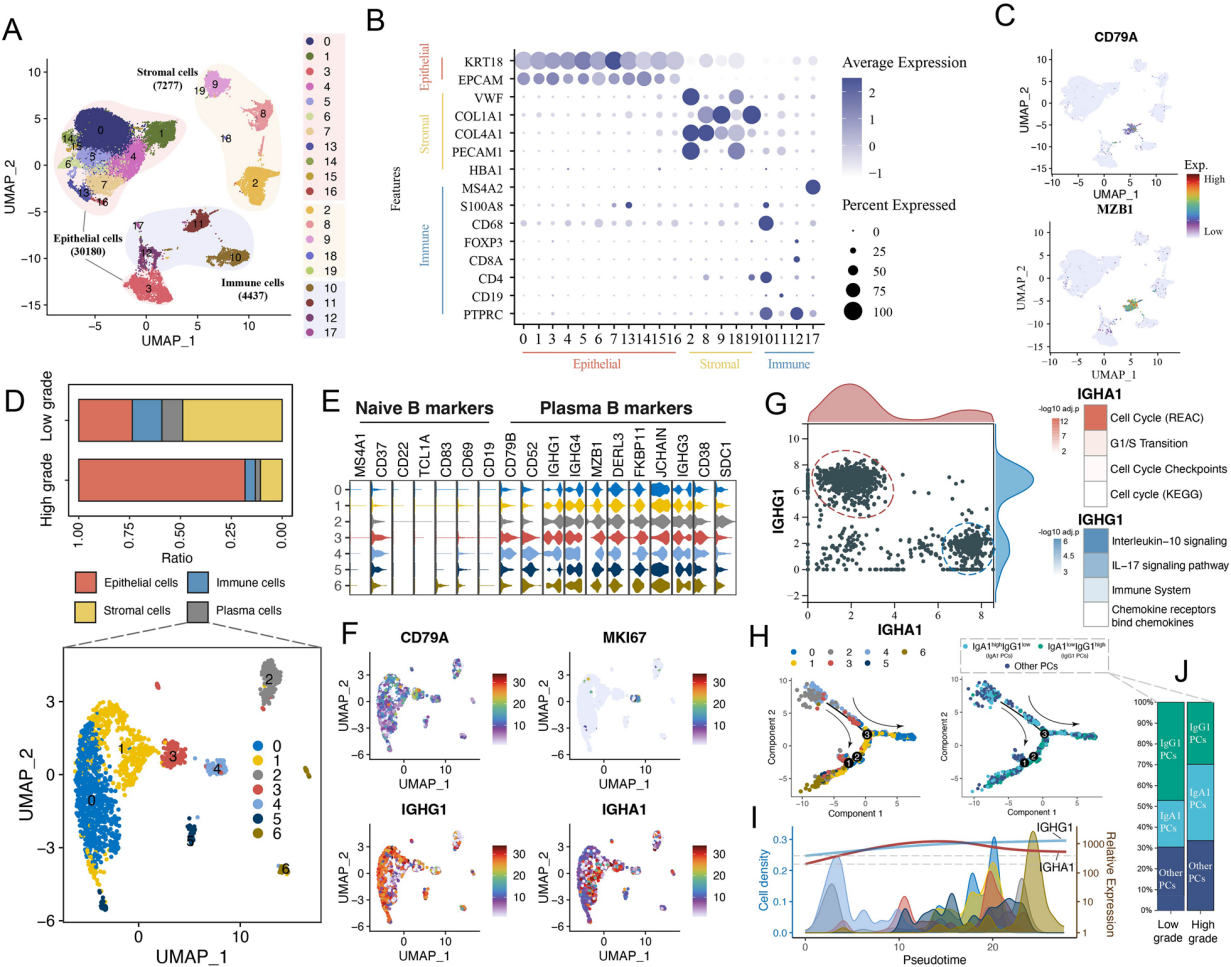
Identification of dominant plasma cell subpopulations by single-cell analysis. **A** UMAP visualization of 41,894 cells across the two low-grade and six high-grade bladder cancer patients. **B** Dot plot showing the expression of specific cell type markers. **C** The expression of plasma cell marker genes. **D** The subclusters of PCs (upper panel) and proportions of cells across the low- and high-grade samples (lower panel). **E** Violin plot showing the expression of naive B- and plasma B-cell marker genes in subclusters of PCs. **F** The expression of selected genes in PCs. **G** Scatter plot showing the independent cell distribution based on the expression of *IGHG1* and *IGHA1* (left panel). The heatmap shows the significantly enriched pathways of IgA1 PCs and IgG1 PCs (right panel). **H** Pseudotime trajectory of subclusters and *IGHG1*/*IGHA1*-associated PCs. **I** Density plot showing the cell distribution, and curve chart showing the expression change of *IGHG1* and *IGHA1* along the pseudotime. **J** Stacked chart showing the distribution of *IGHG1*/*IGHA1*-associated PCs

To further explore the heterogeneity of PCs at the single-cell level, we included an scRNA-seq dataset containing two low-grade and six high-grade BC samples. After quality control and cell filtering, 41,894 cells were obtained, including 30,180 epithelial cells (*KRT18*, *EPCAM*), 7,277 stromal cells (*VWF*, *COL1A1*), and 4,437 immune cells (*PTPRC*, *CD19*, *MS4A2*) (Fig. 3A and B). Among them, Cluster 11 was identified as PCs because of the high expression of *CD79A*, *MZB1* and other PC markers (*SDC1*, *CD79B*, *CD52*, *IGHG1/3/4*, *DERL3*, *FKBP11*, *JCHAIN*, and *CD38*) (Fig. 3C and E). By comparing the proportion of cells between groups, we found a decrease in PCs in high-grade patients compared with low-grade patients (Fig. 3D, upper panel). Next, PCs were further subdivided into seven clusters (Fig. 3D, lower panel) without expression of naive B-cell markers, indicating that these cells were all mature PCs (Fig. 3E). Cluster 4 was identified as plasmablasts due to high expression of the proliferation-related gene *MKI67*, while the other cells mainly expressed *IGHG1* and *IGHA1* (Fig. 3F). Concurrently, other antibody genes (*IGHG2*, *IGHA2*, *IGHM* and *IGHD*) had low or almost no expression (Additional file 1: Figure S2A). After antibody gene-based dimension reduction through PCA, we observed that cells highly expressing *IGHG1* and *IGHA1* constituted almost all PCs, and there was overlap of cells with multiple IgG subtypes [51] (Additional file 1: Figure S2B). The scatter plot also showed a significant distinction between the two cell types (Fig. 3G), suggesting that IgG1 and IgA1 PCs were the dominant PC types in BC.

To further explore their functional characteristics, cells with high *IGHG1* and low *IGHA1* expression were defined as IgG1 PCs, and those with low *IGHG1* and high *IGHA1* expression were defined as IgA1 PCs (Additional file 1: Figure S3). Among them, IgA1 PCs were mainly involved in cell cycle-related pathway regulation (e.g., cell cycle and G1/S transition), while IgG1 PCs were significantly associated with immune responses (e.g., immune system and IL-17 signaling pathway) (Fig. 3G), suggesting that IgG1 PCs may be more likely to be involved in antitumor immune regulation. We further determined the order of differentiation of PCs by pseudotime trajectory analysis; IgA1 PCs were located at the beginning of the trajectory, and IgG1 PCs were located mainly at the end (Fig. 3H). Concurrently, we also observed that *IGHA1* expression decreased at the end of the trajectory, while *IGHG1* increased (Fig. 3I), suggesting a possible class-switched relationship between IgA1 and IgG1 PCs. Interestingly, the differentiation trajectory showed a clear branch at root 3 (Additional file 1: Figure S4), with cells of cell fate 2 presenting high expression of HSP family members and MHC-II-like molecules, which were absent in cell fate 1, implying a difference in the function of PCs with the two different fates.

To explore the effect of IgA1 and IgG1 PCs on cancer progression and patient survival, cell ratio analysis was performed; the results showed an increase in IgA1 PCs and a decrease in IgG1 PCs in patients with high-grade disease (Fig. 3J). However, heterogeneity was observed in the impact of IgA1 and IgG1 PCs on patient survival in different BC subtypes (Additional file 1: Figure S5A). For example, high signature scores of IgG1 predicted better OS in the basal/squamous (Ba/Sq) subtype (log-rank *P* = 0.0011) and luminal unstable (LumU) subtype but worse OS in the luminal papillary (LumP) subtype (log-rank *P* = 0.0075). In contrast, high signature scores of IgA1 PCs predicted better OS in the Ba/Sq subtype (log-rank *P* = 0.006) but tended to predict worse OS in the LumP and LumU subtypes. Although different types of PCs may have different functional roles in different subtypes, IgG1 PCs were dominant in all subtypes (Additional file 1: Figure S5B).

### Identification of six tumor cell states

**Fig. 4:**
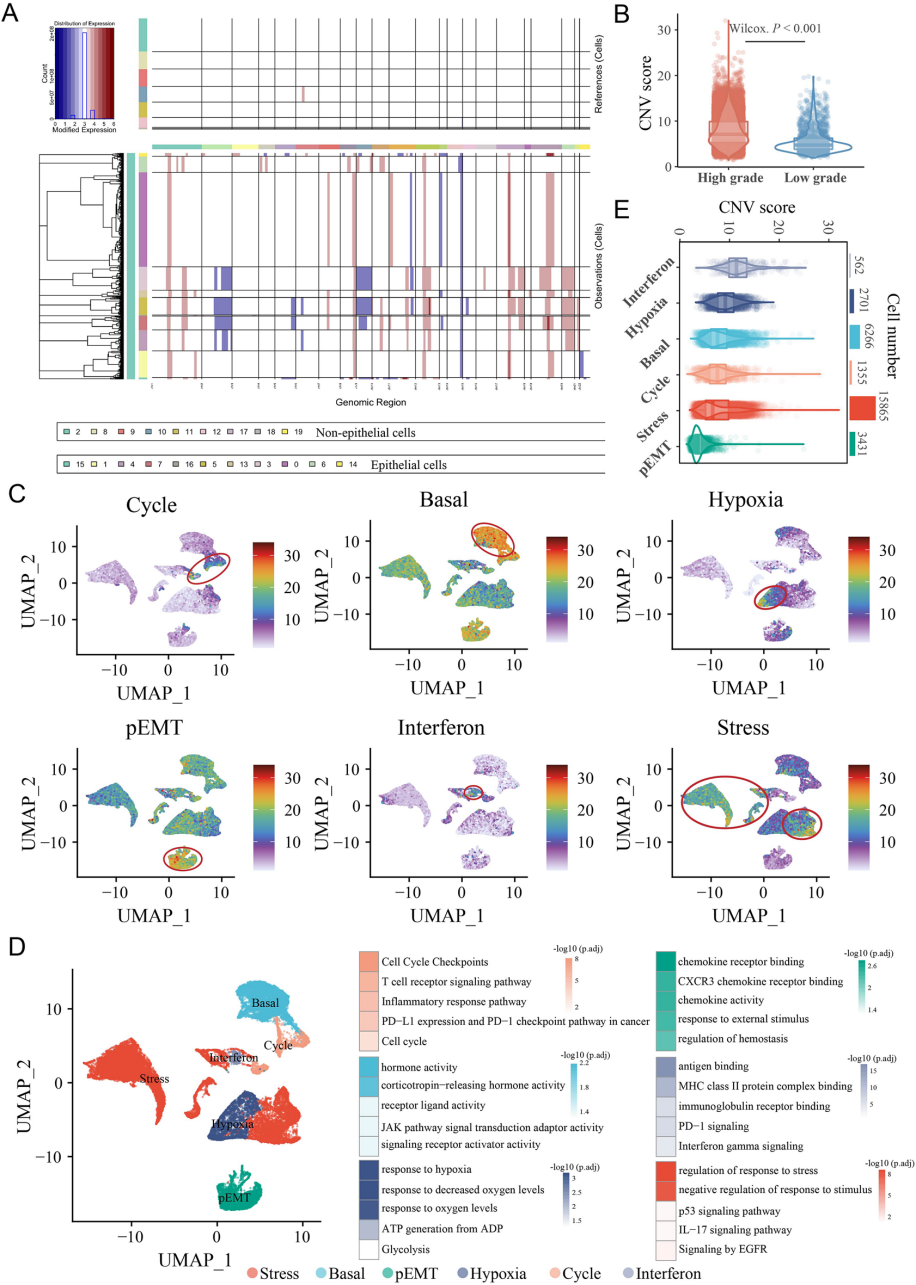
Identification of six cell states by nonnegative matrix factorization (NMF). **A** Epithelial cells had more significant copy number variations (CNVs) of chromosomes than nonepithelial cells. **B** The CNV score was higher in high-grade samples. **C** Six epithelial (tumor) cell state scores calculated by the AUCell R package based on NMF dimension reduction. **D** UMAP visualization of the six tumor cell states (left panel) and significantly enriched pathways (right panel). **E** The CNV scores were different among cells of the six tumor cell states

Based on copy number inference analysis in all single cells, tumor cells came from epithelial cells with significant copy number variation (CNV) (Fig. 4A), and almost all had amplifications in chromosomes 1, 8, 12, 17 and 19 and deletions in chromosome 14. Consistent with previous reports [23], cells from high-grade patients had higher CNV scores (Fig. 4B). Six tumor cell states (cycle, basal, hypoxia, partial epithelial-mesenchymal transition [pEMT], interferon, and stress) in BC were identified and used to assess the intrinsic functional characteristics of tumor cells (Fig. 4C and D). The tumor cells in different states were found to be involved in different regulatory functions (Fig. 4D). For example, cycle-like tumor cells were mainly associated with cell cycle checkpoints, and they may be involved in the T-cell receptor signaling pathway and regulate the PD-L1/PD-1 checkpoint pathway in cancer. Stress-like tumor cells were found to regulate the response to stress and the p53/IL-17 signaling pathway Additionally, it is worth noting that interferon-like tumor cells were found to be involved in the regulation of key immune response processes, such as antigen binding, MHC class II protein complex binding, immunoglobulin receptor binding, and PD-1 signaling. Although interferon-like tumor cells had the lowest cell proportion, they had the highest CNV scores (Fig. 4E).

We then evaluated the impact of different tumor cell states on patient survival (Additional file 1: Figure S6). In the nonimmunotherapy TCGA-BLCA cohort, high basal (Cox *P* = 0.015; *HR* = 1.20, 95% *CI* 1.00–1.40) and pEMT scores (Cox *P* = 0.044; *HR* = 1.20, 95% *CI* 1.00–1.40) were associated with worse OS. In the anti-PD-L1-treated IMvigor210 cohort, high cycle (Cox *P* = 0.012; *HR* = 0.84, 95% *CI* 0.73–0.96) and stress scores (Cox *P* = 0.004; *HR* = 1.30, 95% *CI* 1.10–1.50) were correlated with better and worse OS, respectively.

### The landscape of crosstalk between tumor cells and PCs

We next investigated the communication between all the cells. Clusters 10, 12, and 17 were further defined as myeloid-derived cells (MDCs) (*LYZ*, *C1QB*), T cells (*CD3D*, *CD3E*), and mast cells (*TPSAB1* and *TPSB2*) (Additional file 1: Figure S7A). Among cells, stromal cells and MDCs were generally the strongest signaling senders and receivers, respectively (Additional file 1: Figure S7B). PCs were more likely to receive signals from stromal cells and send signals to T cells and MDCs (Additional file 1: Figure S7C), suggesting that PCs may be important immune mediators regulating tumor microenvironment (TME) intercellular communication in BC.

In the analysis of pathways based on L/R pairs between tumor cells and PCs, midkine (MK) and macrophage migration inhibitory factor (MIF) signaling were the most significantly enriched pathways (Additional file 1: Figure S8A and B). We further found that MK signaling was mainly enriched in tumor cells but not in PCs. In the MIF signaling pathway, tumor cells were the main output cells, especially interferon-like tumor cells (which were also the main signaling cells in all pathways), whereas PCs were the main receiver cells (Additional file 1: Figure S8C–E). Furthermore, it is worth noting that PCs were more likely to receive signals from interferon-like tumor cells and send signals to cycle-like tumor cells in low-grade BC samples (Additional file 1: Figure S9), while this communication pattern was disrupted in high-grade samples, in which PCs received additional signals from pEMT- and hypoxia-like tumor cells and sent more signals to interferon-like tumor cells. Together, these data suggest that the communication pattern between tumor cells and PCs dynamically changes with BC progression.

**Fig. 5:**
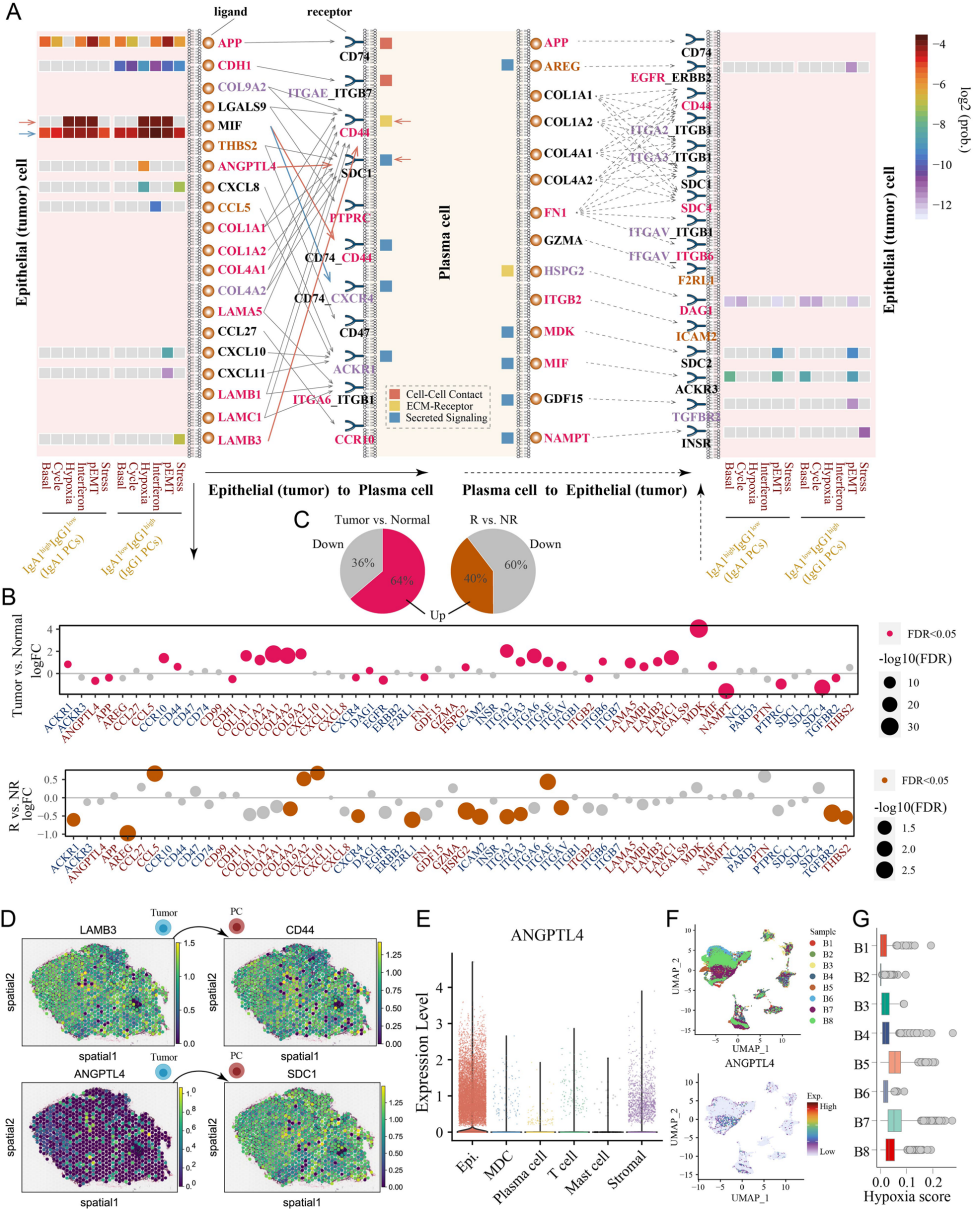
Potential ligand and receptor interactions between PCs and tumor cells. **A** Crosstalk pattern diagram showing signaling from tumor cells to plasma cells and from plasma cells to tumor cells. The internal heatmap shows the communication probability of the selected L/R pair crosstalk between tumor cells of six different states and IgG1 and IgA1 PCs. The differentially expressed L/R pairs between tumor and normal samples from the TCGA-BLCA cohort are marked in red, and those between samples from responding versus nonresponding patients from IMvigor210 are marked in brown; those in both cohorts are marked in purple. The differential expression analysis was performed by the limma R package, and the *P* value was adjusted by the BH method. **B** Bubble plots showing the change in the expression of L/R pairs between tumor and normal samples from the TCGA-BLCA cohort (upper panel) and between patients who responded and did not respond to anti-PD-L1 therapy from the IMvigor210 cohort (lower panel). **C** Pie chart showing the ratio of upregulated to downregulated molecules in the two groups. **D** The expression of selected genes in tissue sections. **E** *ANGPTL4* was mainly expressed in epithelial cells, followed by stromal cells. **F** UMAP plot showing the expression bias of *ANGPTL4* in samples B5 and B7. **G** Box plot showing the high hypoxia score in samples B5 and B7

In the analysis of L/R pairs, the L/R pairs of MIF/(CD74 + CD44 or + CXCR4) and APP/CD74 were the most prominent interactions involving signal transduction from tumor cells to PCs (Fig. 5A). The protumor effects of MIF and APP have been reported in several studies, and their potential association with PCs may explain the additional mechanisms of tumor progression [52–54]. Moreover, in the analysis of the correlation between L/R pairs and patient OS based on the anti-PD-L1 treatment cohort, some L/R pairs mediated signal transduction from hypoxia-like and stress-like tumor cells to IgG1 PCs (such as ANGPTL4/SDC1 and LAMB3/CD44, respectively), demonstrating a significant association with worse patient OS (log-rank *P* < 0.05), suggesting that these L/R pairs may play specific roles in patients receiving ICB therapy (Additional file 1: Figure S10A and S10B). On the other hand, some chemokine signaling-related molecules (such as *CXCL10* and *CXCL11*) were associated with better patient OS (Fig. 5A), and these chemokines secreted by pEMT-like tumor cells may promote the recruitment of IgG1 PCs via ACKR1 [55]. In addition, the expression levels of 58 L/R pairs involved in significant communication between tumor cells and PCs were further investigated. In the TCGA-BLCA cohort, 33 molecules were differentially expressed between tumor and normal samples (*FDR* < 0.05), 64% of which were upregulated in tumor samples (Fig. 5B and C). In the anti-PD-L1 treatment IMvigor210 cohort, 16 molecules were differentially expressed between treatment-responsive and nonresponsive patients, but only 40% were upregulated in responsive patients (Fig. 5B and C). Thus, we speculated that global crosstalk between tumor cells and PCs may promote cancer development and be related to a worse response to ICB therapies.

### Validation of L/R pair expression through spatial transcriptome analysis

To further select reliable L/R pairs between tumor cells and PCs, we assessed their expression levels in tissue sections using spatial transcriptome analysis. Sample GSM5224027 was used for expression assessment. We observed that Cluster 6 was located in a tertiary lymphoid-like structure in BC tissue, and the PC markers *IGHG1* and *IGHA1* also tended to be enriched (Additional file 1: Figure S11). Concurrently, the epithelial cell marker *KRT18* was also found to be significantly expressed, whereas the expression of *EPCAM* was slightly lower. Based on the expression and biological localization analyses of L/R pairs, we identified two L/R pairs (LAMB3/CD44 and ANGPTL4/SDC1) with high reliability between tumor cells and PCs (Fig. 5D and Additional file 1: Figure S11). However, we found low *ANGPTL4* expression in the tissue, which was also validated in the additional sample GSM5224029 (Fig. 5D and Additional file 1: Figure S12), suggesting that ANGPTL4 may be secreted only by some specific tumor cells. We examined the expression of *ANGPTL4* in different cell types and found that it was significantly expressed in epithelial (tumor) cells and to a lesser extent in some stromal cells (Fig. 5E). In the analysis of the sample sources, a significant sample bias of *ANGPTL4* was observed in samples B5 and B7, which could be related to their significant hypoxic levels (Fig. 5F and G). Hypoxic conditions induce ANGPTL4 expression [56]. We speculate that hypoxic tumor cells may secrete ANGPTL4 to communicate with SDC1^+^ PCs, affecting their differentiation, survival, and/or antibody secretion.

### Construction of a risk model based on L/R pairs

**Fig. 6:**
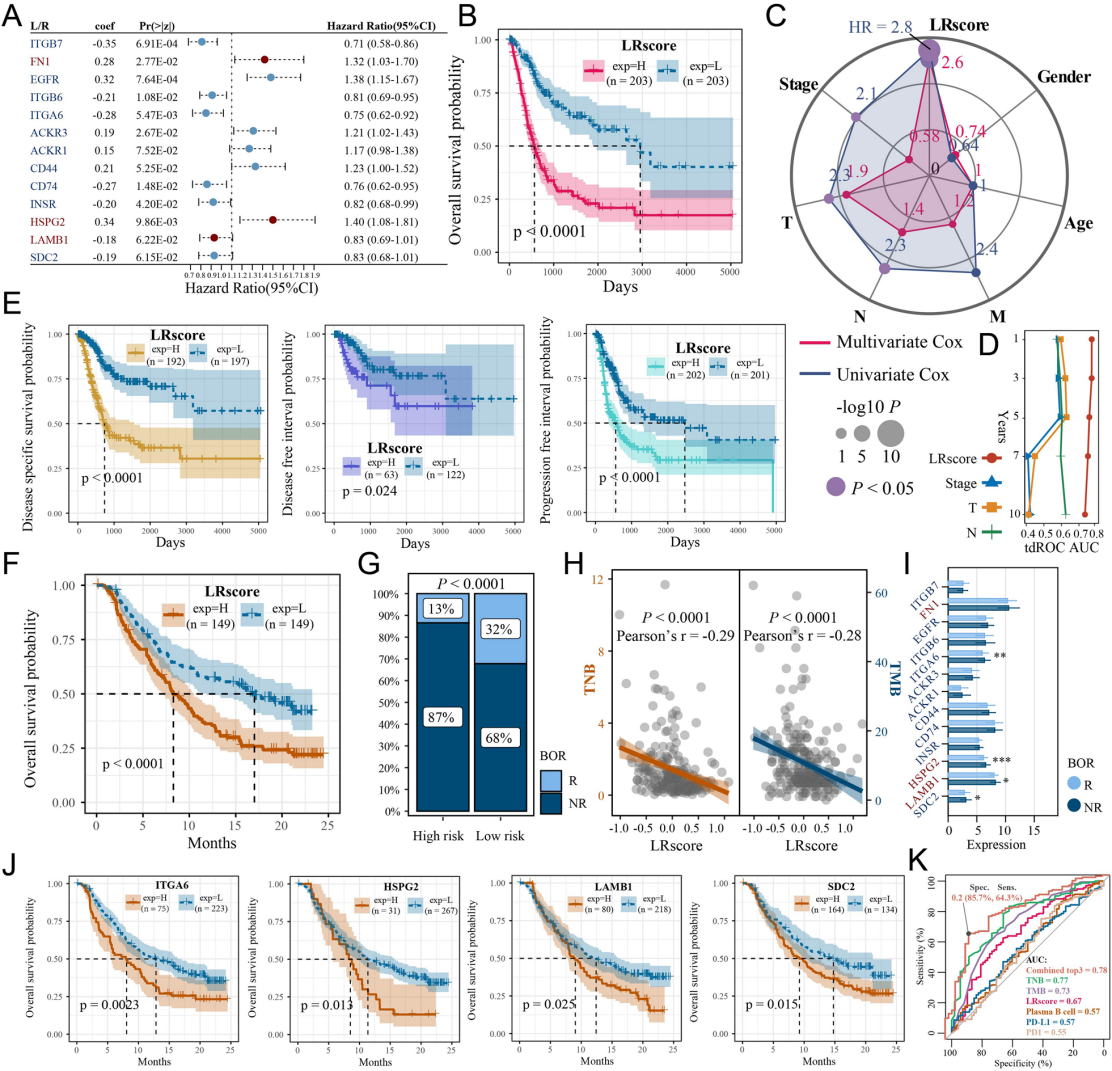
The ligand (L)- and receptor (R)-based risk model LRscore is a good prognostic and immune predictor. **A** Forest plot showing the hazard ratio (HR) of selected L/R pairs based on stepwise Cox regression analysis. **B** Kaplan–Meier curve showing that a high LRscore was associated with poor OS in TCGA-BLCA patients. **C** Radar chart showing that the LRscore was significantly correlated with risk according to univariate and multivariate Cox regression analyses. **D** The LRscore had the highest AUC and stability for predicting 1-, 3-, 5-, 7-, and 10-year survival. **E** Kaplan–Meier curves showing that a high LRscore was associated with poor DSS, DFS and PFS in TCGA-BLCA patients. **F** Kaplan–Meier curve showing that a high LRscore was associated with poor OS in IMvigor210 patients. **G** Stacked histogram showing that the high-risk group had a lower percentage of responding patients than the low-risk group. **H** The LRscore was negatively correlated with TMB and TNB in the anti-PD-L1 treatment cohort. **I** Three model genes demonstrated differential expression between the response (R) and nonresponse (NR) groups in the anti-PD-L1 treatment cohort. Wilcoxon test; *P < 0.05; **P < 0.01; ***P < 0.001. **J** Kaplan–Meier curves showing that *ITGA6*, *HSPG2*, *LAMB1* and *SDC2* are associated with the OS of IMvigor210 patients. **K** Receiver operator characteristic (ROC) curve showing the area under the curve (AUC) for different indicators used to identify patients with R or NR

We further quantified the crosstalk patterns between tumor cells and PCs and assessed their association with patient survival and immunotherapy. Based on the TCGA-BLCA cohort, we selected 13 molecules from 58 L/R pairs involved in significant communication between tumor cells and PCs to construct a risk model (Fig. 6A), and the risk score was calculated according to the following equation:

$$\mathrm{LRscore}= -0.35*{Exp.}_{ITGB7}+0.28*{Exp.}_{FN1}+0.32*{Exp.}_{EGFR}-0.21*{Exp.}_{ITGB6}-0.28*{Exp.}_{ITGA6}+0.19*{Exp.}_{ACKR3}+0.15*{Exp.}_{ACKR1}+0.21*{Exp.}_{CD44}-0.27*{Exp.}_{CD74}-0.20*{Exp.}_{INSR}+0.34*{Exp.}_{HSPG2}-0.18*{Exp.}_{LAMB1}-0.19*{Exp.}_{SDC2}$$

We found that patients in the high LR score group had significantly worse OS (log-rank *P* < 0.0001) in the univariate Cox analysis (Fig. 6B). Stage, T stage, and N stage were also significantly associated with OS in the univariate Cox analysis (Cox *P* < 0.05, *HR* > 2) (Fig. 6C), while the LRscore was the only independent predictor for patients with BC (Cox *P* = 7.30E-08, *HR* = 2.6) after removing the confounding factors in the multivariate Cox analysis (Fig. 6C). The area under the tdROC curve (AUC) for 1-, 3-, 5-, 7-, and 10-year survival showed that the LRscore was a more stable and effective indicator than stage, T stage, and N stage (Fig. 6D). Moreover, a high LRscore was also associated with worse DSS (log-rank *P* < 0.0001), DFS (log-rank *P* = 0.024), and PFS (log-rank *P* < 0.0001) in BC patients (Fig. 6E), suggesting that the LRscore also has the potential to predict patient relapse and progression.

We further evaluated the effect of the LRscore on patient survival in the anti-PD-L1 treatment cohort, in which a high LRscore also predicted worse OS (log-rank *P* < 0.0001) (Fig. 6F). Concurrently, fewer patients responded to treatment in the high-risk group (high-risk vs*.* low-risk = 13% vs. 32%) (Fig. 6G), suggesting that the LRscore may be associated with poorer patient response to ICB therapies. This finding was further validated by the significant negative correlations of the LRscore with TNB (*P* < 0.0001, Pearson’s *r* = −0.29) and TMB (*P* < 0.0001, Pearson’s *r* = −0.28) (Fig. 6H). Moreover, we found that the expression of the model molecules *ITGA6*, *HSPG2*, *LAMB1*, and *SDC2* was lower in responding patients (Wilcoxon test, *P* < 0.05), and low expression of these molecules was associated with poorer OS (log-rank *P* < 0.05) (Fig. 6I and J). These model molecules may be the main contributors to the success of the model in predicting the immune response in the anti-PD-L1 therapy cohort. Finally, we evaluated the predictive effect of the LRscore versus other immunotherapy-related predictors (TNB, TMB, PD-L1, PD1, and PCs) (Fig. 6K). The combination of the LRscore with TNB and TMB obtained the highest AUC (0.78) with a diagnostic specificity of 85.7%, and thus could be used to avoid excessive immunotherapy in patients.

### Assessment of the risk model

**Fig. 7:**
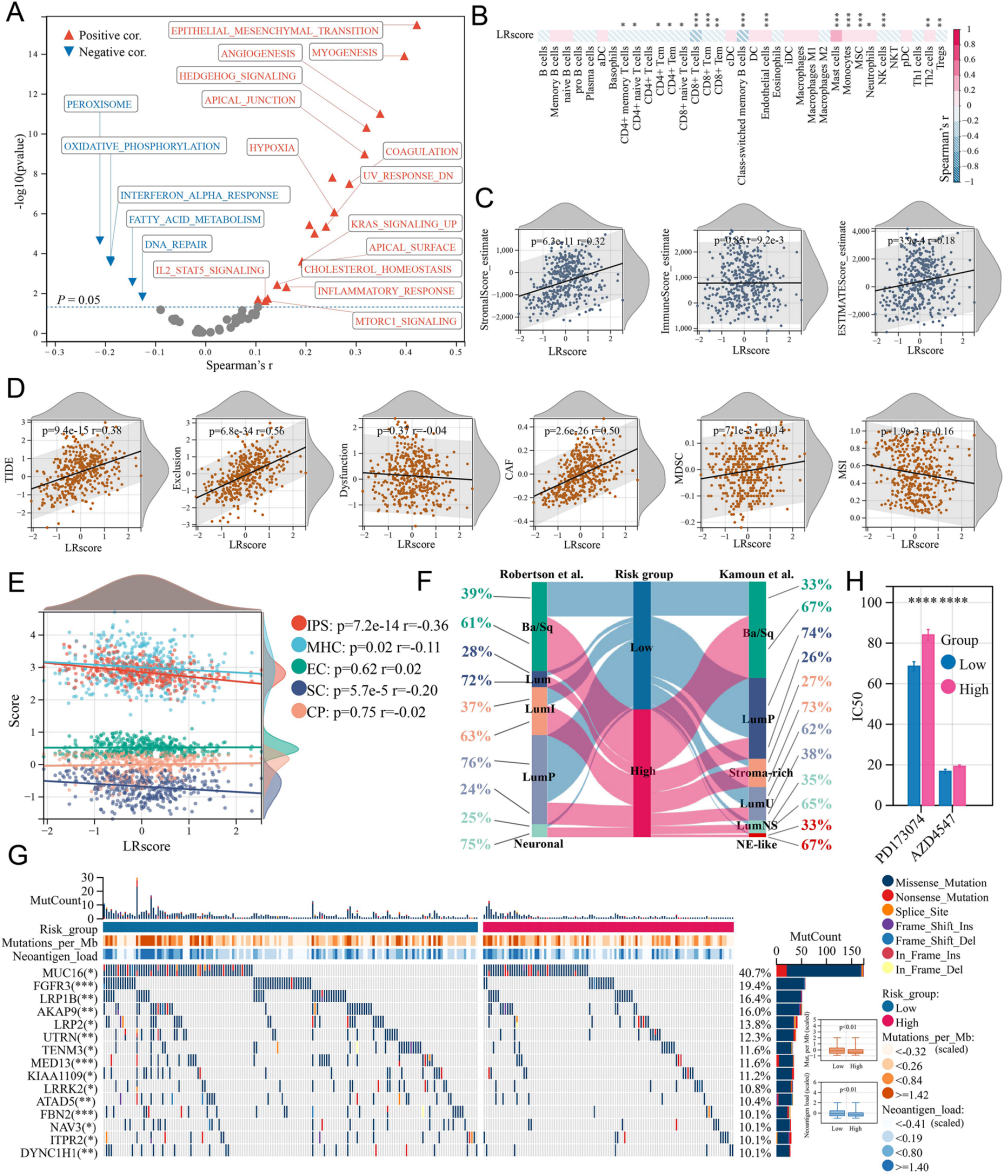
Characterization of the relationship of the LRscore with cancer progression, the microenvironment, cancer subtype and somatic mutation. **A** Volcano plot showing that the LRscore was positively correlated with the majority of cancer hallmarks. In the Spearman analysis, a cancer hallmark with *P* < 0.05 is marked in red (positive correlate) or blue (negative correlate). **B** Heatmap showing the association between the LRscore and immune cell infiltration level inferred based on xCell analysis. Spearman analysis; *P < 0.05; **P < 0.01; ***P < 0.001. **C-E** The LRscore was correlated with some indicators of the microenvironment, immune dysfunction/exclusion, and immune phenotype (Spearman analysis). **F** Sankey chart showing the distribution of different LRscore risk patients across different molecular subtypes of bladder cancer. **G** Oncoplot showing differences in TMB, TNB and mutated genes between the high- and low-risk groups. **H** The standard error bars indicate the IC50 values for the two *FGFR3*-targeted drugs in the low- and high-risk groups, with a lower IC50 typically predicting higher drug sensitivity. Wilcoxon test; ****P < 0.0001

To better analyze the features of the LR risk model, we first evaluated the correlation between the LRscore and cancer hallmarks based on the TCGA-BLCA cohort (Fig. 7A). The LRscore was positively correlated with most cancer hallmarks (*P* < 0.05, Spearman's *r* > 0.1), such as epithelial-mesenchymal transition, angiogenesis, hedgehog signaling, and hypoxia. Immune cell infiltration analysis showed that the LRscore was negatively correlated (*P* < 0.05, Spearman's *r* < −0.1) with the majority of tumor- suppressive T-cell types (Fig. 7B) and the IPS (*P* = 7.2E-14, Spearman's *r* = −0.36) (Fig. 7E). The IPS is an immunogenicity evaluation index, and a higher IPS indicates better immunotherapy performance. However, the LRscore was positively correlated with endothelial cells and the stromal score but not with effector cells (ECs), immune checkpoints (CPs), or the global immune score (Fig. 7C and E). More importantly, a significant positive association between the LRscore and TIDE score (*P* = 9.4E-15, Spearman's *r* = 0.38) was also observed, and this association was mainly derived from exclusion (*P* = 6.8E-34, Spearman's *r* = 0.56) and CAFs (*P* = 2.6E-26, Spearman's *r* = 0.50) rather than dysfunction (*P* = 0.37, Spearman's *r* = −0.04) (Fig. 7D). These data suggest that an immunosuppressed state reflected by the LRscore may mainly result from resistance of the tumor stroma to immune infiltration.

In addition, patients in the low-risk group were mostly categorized into the classic Robertson-LumP and consensus Kamoun-LumP molecular subtypes of BC, whereas the high-risk group was associated with malignant subtypes such as the Ba/Sq, Robertson-Neuronal, and Kamoun-NE-like subtypes (Fig. 7F). Remarkably, the LumP subtype showed a high *FGFR3* mutation frequency in the low-risk group (Chi-square test, *P* < 0.001) (Fig. 7G). Moreover, we performed a drug sensitivity analysis based on the GDSC drug database for different risk groups, and patients in the low-risk group showed higher sensitivity to *FGFR3*-targeted drugs (such as PD173074 and AZD4547) (Fig. 7H). *FGFR3* could be a drug target for patients in the low-risk group. Overall, these data suggest that a high LRscore predicts high immune exclusion and low immunogenicity in patients, suggesting poorer immunotherapy response and OS, but patients with a low LRscore may have better immune and targeted therapy outcomes.

## Discussion

Over the past few years, improvements have been made in the treatment of BC patients due to the increased understanding of tumor molecular profiles and the development of ICB therapies. However, for the majority of patients (who do not respond to therapy), current efforts are still inadequate. Previous studies have reported a dual role of PCs in protumor and antitumor regulation [57]. However, the function of PCs and the communication between PCs and tumor cells are not fully understood in BC patients. Here, we explored the crosstalk between the major functional subtypes of PCs (IgG1 and IgA1) and tumor cells of six different states in BC patients and confirmed the impact of these crosstalk patterns on patient prognosis and immunotherapy efficacy.

The effects of antitumor antibodies on oncological outcomes partly depend on the antibody isotype. Generally, IgG1 exerts antitumor effects by enhancing T-cell responses, while IgA exerts protumor effects by inducing IL-10 release from myeloid cells [58]. In this study, IgG1 PCs were predominant in the TIME and showed a significant association with inflammatory response pathways but had a lower infiltration level in high-grade BC. Instead, IgA1 PCs had higher infiltration in high-grade BC, and pseudotime analysis showed the existence of conversion between IgG1 and IgA1 PCs. These results further suggest an important role for PC isotype switching in BC. However, we also found that the cancer-suppressing and cancer-promoting functions of IgG1 and IgA1 PCs may be heterogeneous across BC subtypes, as reflected in their different associations with patient survival. This may be due to the inconsistent expression of complement factors, such as C3a and C5a, and differences in polymer Ig receptor (pIgR) abundance between different subtypes of BC. In settings with high levels of complement components, IgG1 can also play a protumor role [59], while IgA can activate CD8^+^ T cells by binding to pIgR in tumor cells [60]. Thus, the detailed functions of different PC types in the context of BC require further confirmation in the future.

Dynamic interactions and crosstalk between B cells and other cells, including T cells and MDCs, profoundly influence the immune response to tumors [61, 62]. In this study, we observed that PCs, as effector B cells, were more likely to send signals to T cells and MDCs. However, significant crosstalk between PCs and tumor cells also existed according to the L/R pair analysis, and most of the interactions were derived from secretion of signaling molecules rather than direct cell‒cell contact. Specifically, tumor cells were generally the source cells that secreted the signaling factors. On the one hand, tumor cells may act on PCs by secreting oncogenic molecules (ANGPTL4 or LAMB3 [63, 64]) to inhibit their antitumor function or promote isotype switching of protumor subtypes. On the other hand, chemokine-related pathways (such as chemokine receptor binding to chemokines, chemokine receptor binding, and chemokine activity) were enriched in IgG1 PCs (Fig. 3G) and pEMT-like tumor cells (Fig. 4D). pEMT-like tumor cells were prone to recruit PCs by secreting cytokines such as CXCL10 or CXCL11 (Fig. 5A). CXCL10 can bind CXCR3 and signal via extracellular signal-regulated kinase to cause B cells to transition into protumor IgG-producing PCs [65].

Unlike B-cell-T-cell interactions, which enhance antitumor immune responses [62], signal transduction from tumor cells to PCs was mostly unfavorable and was associated with nonresponse to immunotherapy; this is not surprising since most L/R pairs were usually highly expressed in BC patients who did not respond to immunotherapy (Fig. 5C). In terms of specific L/R pairs, based on the spatial transcriptome expression assessment, stress-like tumor cells were likely to interact with CD44^+^ IgG1 PCs by secreting LAMB3. Stress-like cancer cells have higher tumor-seeding capabilities [66], and *LAMB3,* as a key gene in stress-like tumor cells, has been reported to encode one of the heterotrimeric glycoproteins of laminin-5 (LN5), which promotes tumor invasion and metastasis [67, 68]. Furthermore, tumor cells can secrete multiple ligands of CD44, including osteopontin and hyaluronic acid, to regulate the homing, activation, maturation, and proliferation of immune cells [69, 70]. Thus, stress-like tumor cells may suppress the antitumor function of CD44^+^ IgG1 PCs and promote tumor progression by secreting LAMB3.

SDC1, also known as CD138, is a marker for plasma cells that is closely associated with immunotherapy responses and survival in cancer patients [71]. The accumulation and differentiation of SDC1/CD138^+^ IgG-producing PCs can be modulated by tumor-associated neutrophils and myeloid-derived suppressor cells in a BAFF- or STAT3-dependent manner [20]. In this study, we identified that hypoxia-like tumor cells may also act on SDC1/CD138^+^ IgG1 PCs by secreting ANGPTL4. ANGPTL4 is a known angiogenic factor that can be directly induced by HIF-1α in tumor cells under hypoxic conditions [72, 73], and its expression is significantly increased in BC patients [74]. In conclusion, a thorough understanding of crosstalk between distinct subtypes of tumor cells and PCs is critical for improving ICI efficacy in BC.

This study has some limitations. First, the final number of PCs obtained for the single-cell analysis was limited, and thus the cell types and transition trajectory may be biased owing to the lack of suitable and sufficient single-cell samples. Second, the evaluation of cell interactions was mainly based on mRNA profiles, and analyses to assess relevant proteomic information, such as immunohistochemistry, immunofluorescence staining, and cellular localization assessment, will be helpful for further evaluation of their communication capacity. Finally, and importantly, our study only revealed the communication potential of PCs and tumor cells, but the specific mechanisms and functions of this crosstalk are still unknown, and further related research needs to be carried out.

## Conclusions

In summary, by integrating scRNA-seq, bulk RNA-seq, and spatial transcriptome data, we systematically characterized the crosstalk patterns between tumor cells and PCs in BC and quantified the potential impact of this crosstalk on patient survival and response to immunotherapy. The LRscore risk model based on L/R pairs can be used to predict clinical risk and immune response.

## Supplementary Information

**Additional file 1: Figure S1.** Immune cells associated with patient overall survival risk in the TCGA-BLCA **(A)** and IMvigor210 cohorts **(B)** based on Cox regression analysis. **Figure S2.** Expression of antibody-related genes in PCs. **(A)** Box plot showing the expression of IgG-, IgA-, IgM-, and IgD-associated genes in PCs (Kruskal‒Wallis test). **(B)** UMAP plot of antibody-related gene expression showing that IgG1 and IgA1 PCs are the dominant PCs based on dimension reduction and PCA with selected genes. **Figure S3.** PCs were classified into four types based on the median expression of *IGHG1* and *IGHA1*. **Figure S4.** Branch point analysis based on the pseudotime trajectory. **Figure S5.** Association of IgG1 and IgA1 PCs with different subtypes of bladder cancer. **(A)** Heterogeneous relationship of IgG1 and IgA1 PCs with survival in patients with different bladder cancer subtypes. **(B)** Abundance of IgG1 and IgA1 PCs inferred by ssGSEA in different subtypes of bladder cancer samples. Wilcoxon test; *, *P* < 0.05; **, *P* < 0.01; ***, *P* < 0.001; ****, *P* < 0.0001. **Figure S6.** Tumor cell states associated with patient overall survival risk in the TCGA-BLCA **(A)** and IMvigor210 cohorts **(B)** based on Cox regression analysis. **Figure S7.** Cell communication analysis for all cell types. **(A)** The marker gene expression of T cells, myeloid-derived cells (MDCs), and mast cells. **(B)** Dot plot showing the incoming and outgoing signal strength in different cell types. **(C)** Crosstalk networks showing that PCs tended to send signals to T cells and MDCs but receive signals from stromal cells. **Figure S8.** Signaling pathways enriched in plasma cell-tumor cell communication. **(A&B)** Heatmap showing the signaling pathways enriched for each cell type in both incoming and outgoing signaling patterns. **(C-E)** Dot plots showing the strengths of incoming and outgoing interactions of each cell type in the MK/MIF signaling and all signaling pathways. **Figure S9.** Comparison of PC and tumor cell crosstalk between low- and high-grade bladder cancer samples. **Figure S10.** Survival analysis based on ligands and receptors. **(A)** Overall survival analysis based on selected L/R pairs between tumor cells and PCs in the anti-PD-L1 treatment IMvigor210 cohort. **(B)** Overall survival analysis based on selected L/R pairs between tumor cells and IgG1 PCs in the anti-PD-L1 treatment IMvigor210 cohort. **Figure S11.** Spatial transcriptome cell clustering and assessment of selected gene expression based on sample GSM5224027. **Figure S12.** Spatial transcriptome cell clustering and assessment of selected gene expression based on the validation sample GSM5224029.

**Additional file 2: Table S1.** IgG1 and IgA1 dominated plasma cell marker genes (top 100).

**Additional file 3: Table S2.** Marker genes of six tumor cell states clusters in bladder cancer (top 100).

**Additional file 4: Table S3.** All ligands and receptors between tumor cells and plasma cells.

## Data Availability

The original data presented in the study are included in the article/Supplementary Materials and further inquiries can be directed to the corresponding authors.
