# Supplementary material for: The potential crosstalk between tumor and plasma cells and its association with clinical outcome and immunotherapy response in bladder cancer
Source: J Transl Med. 2023 May 3;21:298. doi: 10.1186/s12967-023-04151-1 (PMC10155334; doi:10.1186/s12967-023-04151-1)
Supplement: Supplementary file 1 — Additional file 1: Figure S1. Immune cells associated with patient overall survival risk in the TCGA-BLCA (A) and IMvigor210 cohorts (B) based on Cox regression analysis. Figure S2. Expression of antibody-related genes in PCs. (A) Box plot showing the expression of IgG-, IgA-, IgM-, and IgD-associated genes in PCs (Kruskal‒Wallis test). (B) UMAP plot of antibody-related gene expression showing that IgG1 and IgA1 PCs are the dominant PCs based on dimension reduction and PCA with selected genes. Figure S3. PCs were classified into four types based on the median expression of IGHG1 and IGHA1. Figure S4. Branch point analysis based on the pseudotime trajectory. Figure S5. Association of IgG1 and IgA1 PCs with different subtypes of bladder cancer. (A) Heterogeneous relationship of IgG1 and IgA1 PCs with survival in patients with different bladder cancer subtypes. (B) Abundance of IgG1 and IgA1 PCs inferred by ssGSEA in different subtypes of bladder cancer samples. Wilcoxon test; *, P < 0.05; **, P < 0.01; ***, P < 0.001; ****, P < 0.0001. Figure S6. Tumor cell states associated with patient overall survival risk in the TCGA-BLCA (A) and IMvigor210 cohorts (B) based on Cox regression analysis. Figure S7. Cell communication analysis for all cell types. (A) The marker gene expression of T cells, myeloid-derived cells (MDCs), and mast cells. (B) Dot plot showing the incoming and outgoing signal strength in different cell types. (C) Crosstalk networks showing that PCs tended to send signals to T cells and MDCs but receive signals from stromal cells. Figure S8. Signaling pathways enriched in plasma cell-tumor cell communication. (A&B) Heatmap showing the signaling pathways enriched for each cell type in both incoming and outgoing signaling patterns. (C-E) Dot plots showing the strengths of incoming and outgoing interactions of each cell type in the MK/MIF signaling and all signaling pathways. Figure S9. Comparison of PC and tumor cell crosstalk between low- and high-grade blad [file 12967_2023_4151_MOESM1_ESM.docx]

***Additional file***

**Figure S1:** Immune cells associated with patient overall survival risk in TCGA-BLCA and IMvigor210 cohorts based on Cox regression analysis.

**Figure S2:** Expression of antibody-related genes in PCs.

**Figure S3:** PCs were classified into four types based on the median expression of IGHG1 and IGHA1.

**Figure S4:** Branch point analysis based on pseudotime trajectory.

**Figure S5:** Association of IgG1 and IgA1 PCs with different subtypes of bladder cancer.

**Figure S6:** Tumor cell states associated with patient overall survival risk in TCGA-BLCA and IMvigor210 cohorts based on Cox regression analysis.

**Figure S7:** Cell communication analysis for all cell types.

**Figure S8:** Signaling pathways enriched in plasma cell-tumor cell communication.

**Figure S9:** Comparison of PCs and tumor cell crosstalk in low- and high-grade bladder cancer samples

**Figure S10:** Survival analysis for ligand and receptor.

**Figure S11:** Spatial transcriptome cell clustering and expression assessment of selected genes based on sample GSM5224027.

**Figure S12:** Spatial transcriptome cell clustering and expression assessment of selected genes based on validation sample GSM5224029.

**
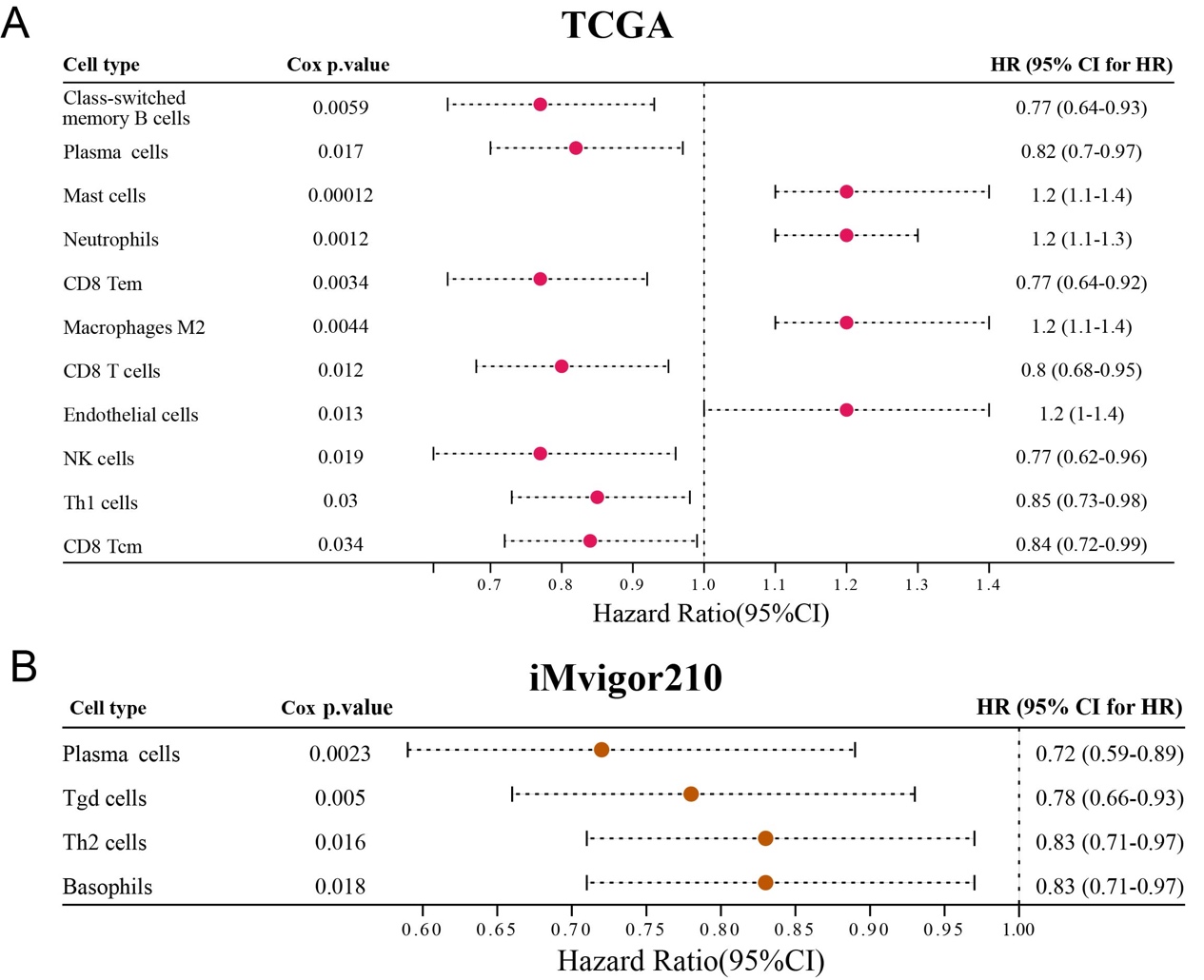
**

**FIGURE S1|** Immune cells associated with patient overall survival risk in TCGA-BLCA **(A)** and IMvigor210 cohorts **(B)** based on Cox regression analysis.


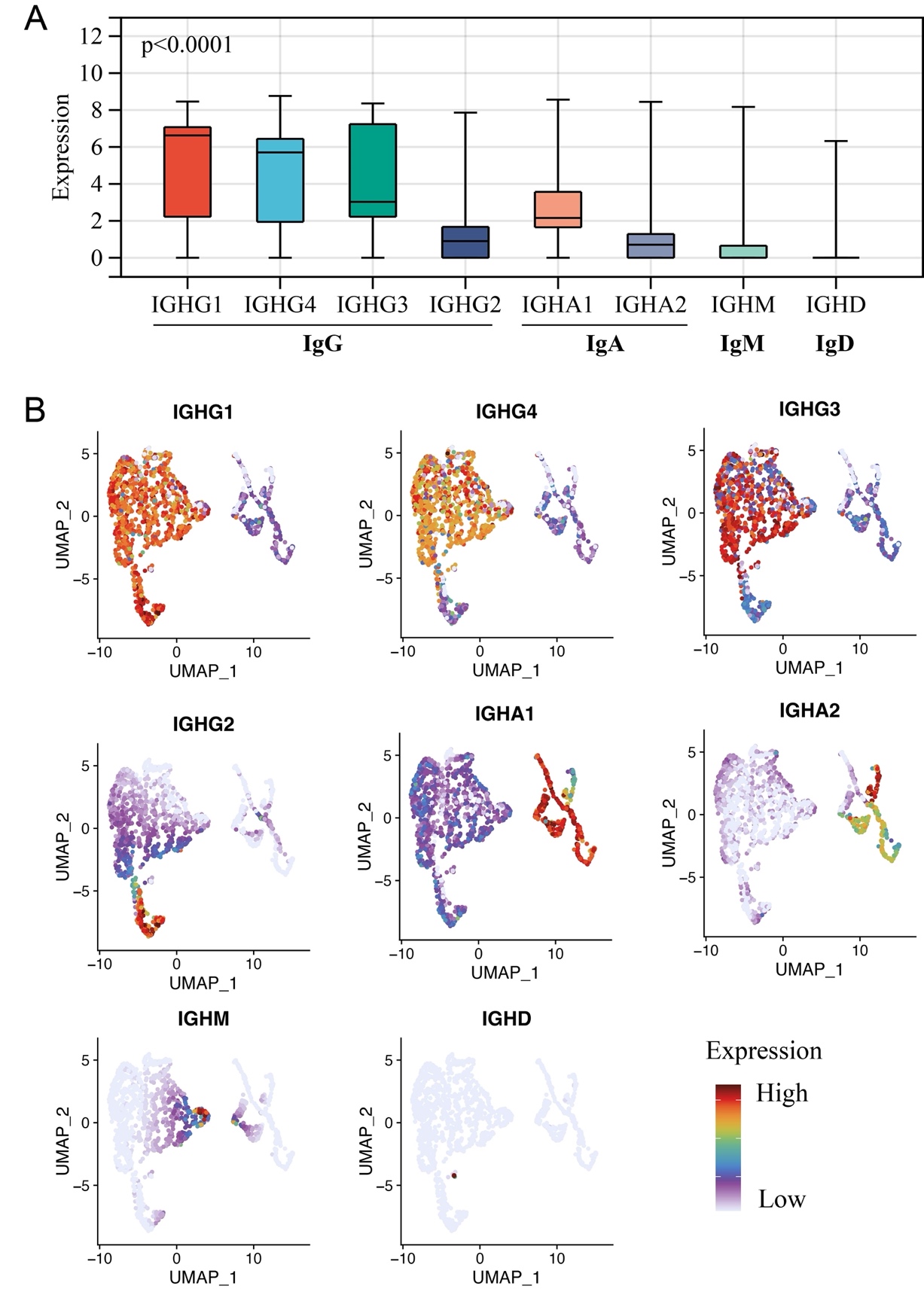


**FIGURE S2|** Expression of antibody-related genes in PCs. **(A)** Box plot shows the expression of IgG-, IgA-, IgM-, and IgD-associated genes in PCs. Kruskal-Wallis test. **(B)** UMAP visualization of antibody-related genes expression showing IgG1 and IgA1 cells are the dominated PCs based on dimension reduction of PCA with selected genes.

**
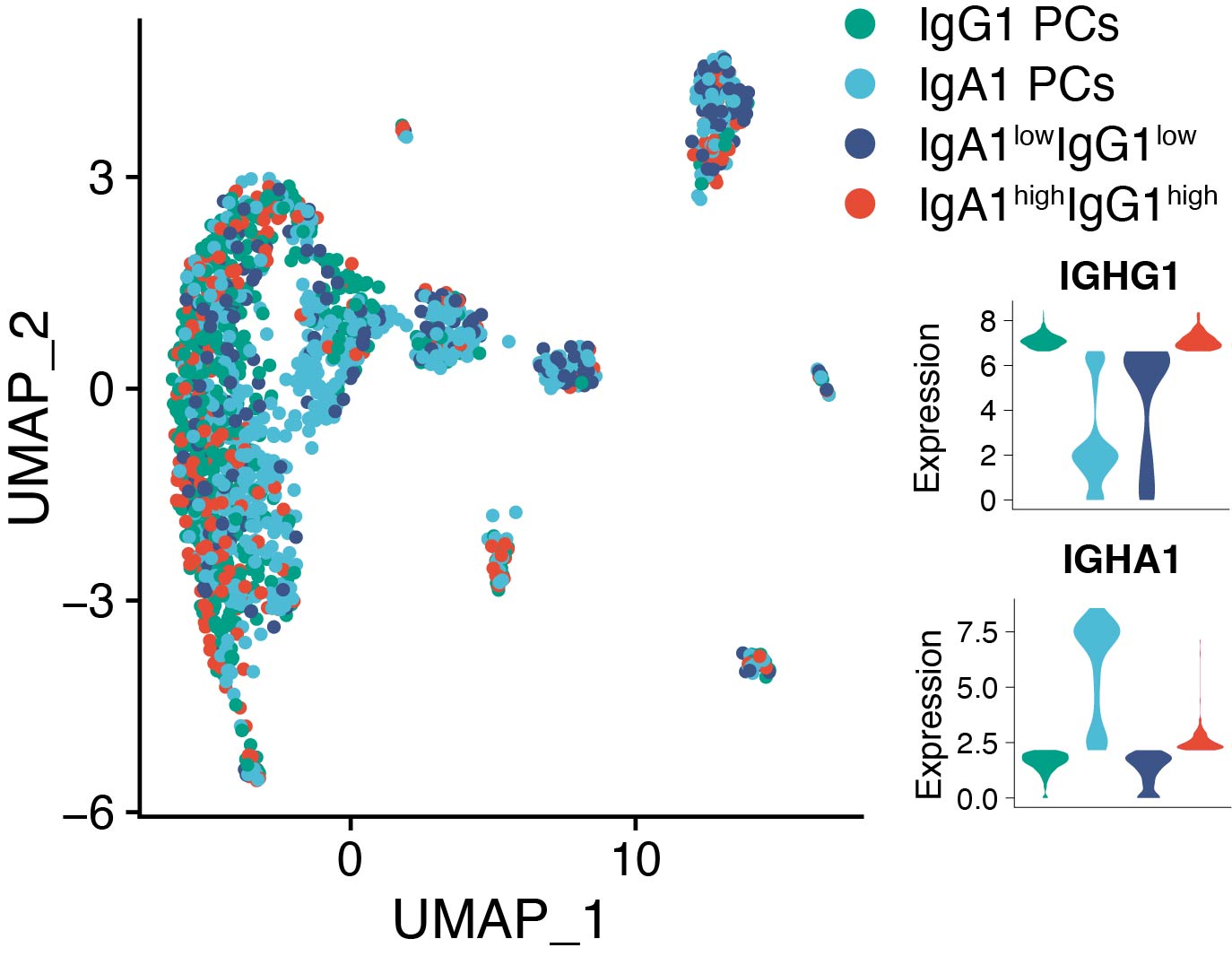
**

**FIGURE S3|** PCs were classified into four types based on the median expression of *IGHG1* and *IGHA1*.

**
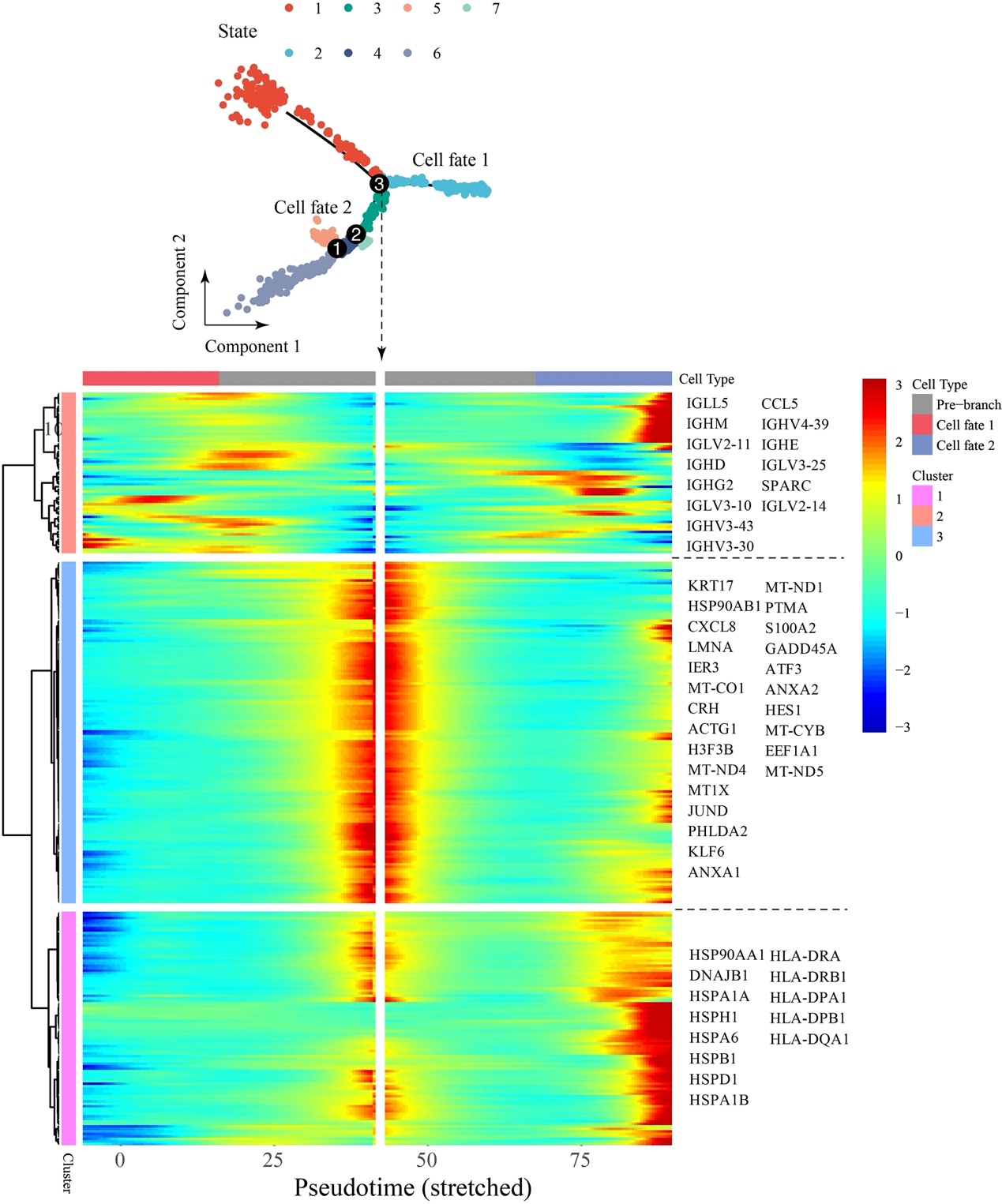
**

**FIGURE S4|** Branch point analysis based on pseudotime trajectory.

**
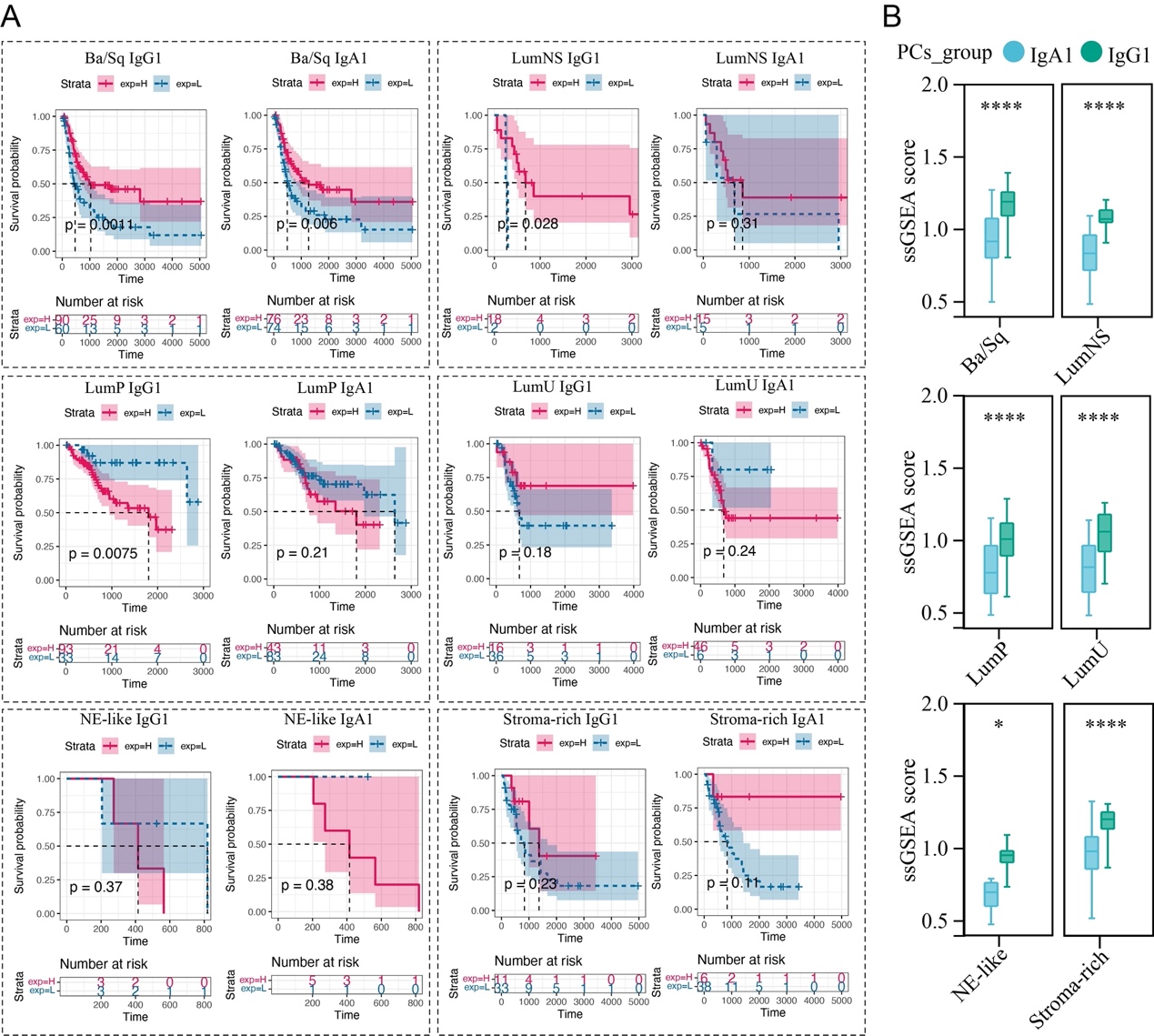
**

**FIGURE S5|** Association of IgG1 and IgA1 PCs with different subtypes of bladder cancer. **(A)** Heterogeneous survival relevance of IgG1 and IgA1 PCs in patients with different bladder cancer subtypes. **(B)** Abundance of IgG1 and IgA1 PCs in different subtypes of bladder cancer samples inferred by ssGSEA. Wilcoxon test; *, *P* < 0.05; **, *P* < 0.01; ***, *P* < 0.001; ****, *P* < 0.0001.

**
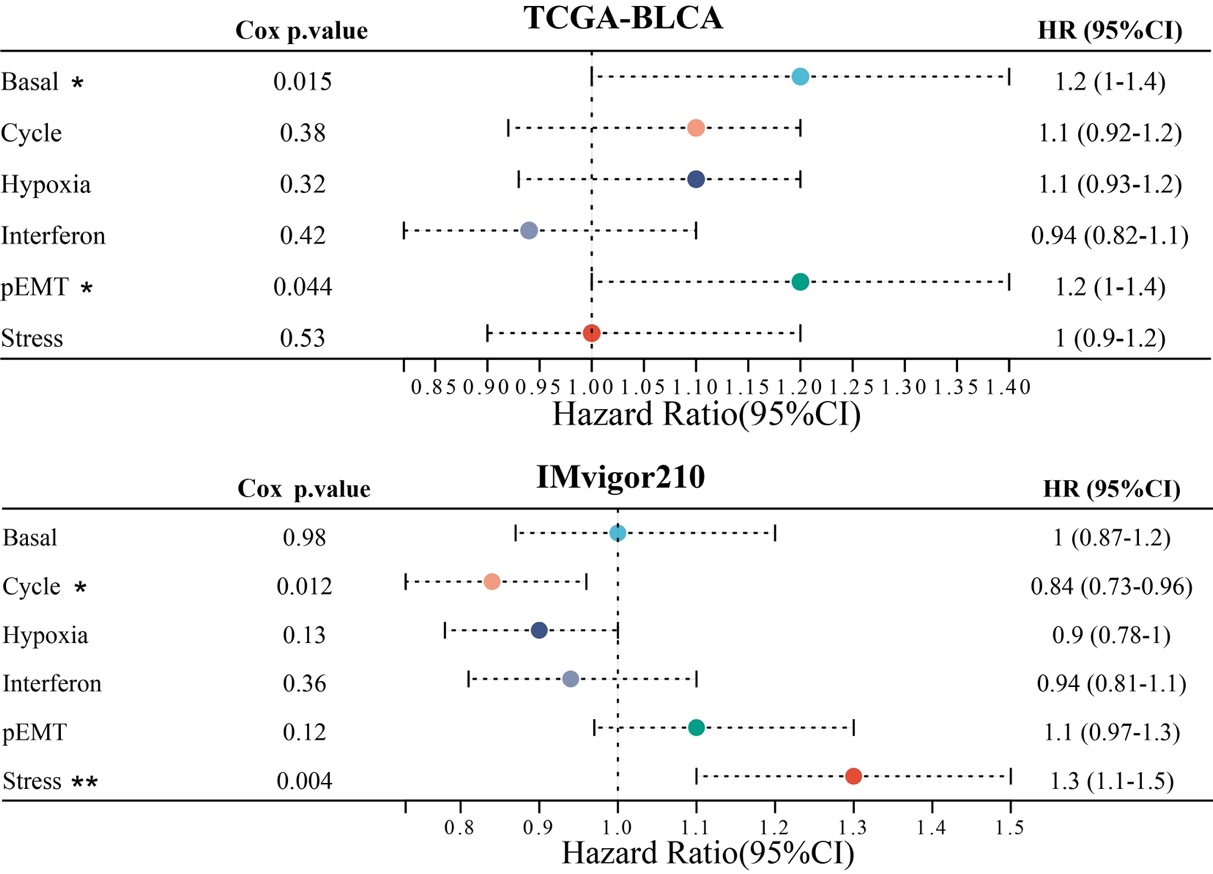
**

**FIGURE S6|** Tumor cell states associated with patient overall survival risk in TCGA-BLCA **(A)** and IMvigor210 cohorts **(B)** based on Cox regression analysis.

**
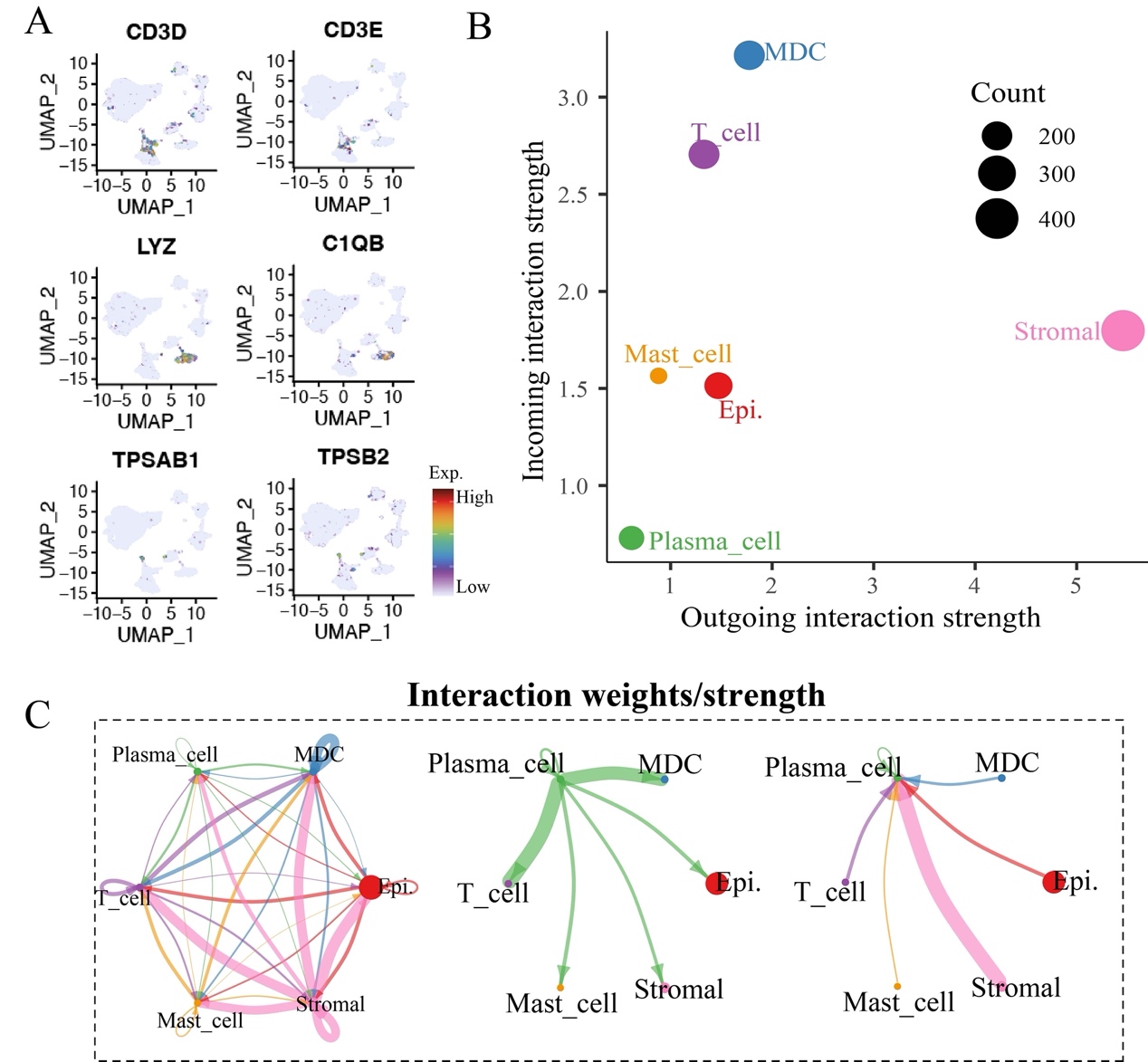
**

**FIGURE S7|** Cell communication analysis for all cell types. **(A)** The marker genes expression of T cell, myeloid-derived cell (MDC), and mast cell. **(B)** Dot plot shows the incoming and outgoing signal strength in different cell types. **(C)** Crosstalk networks show PCs tend to send signals to T cells and MDCs but receive signals from stromal cells.

**
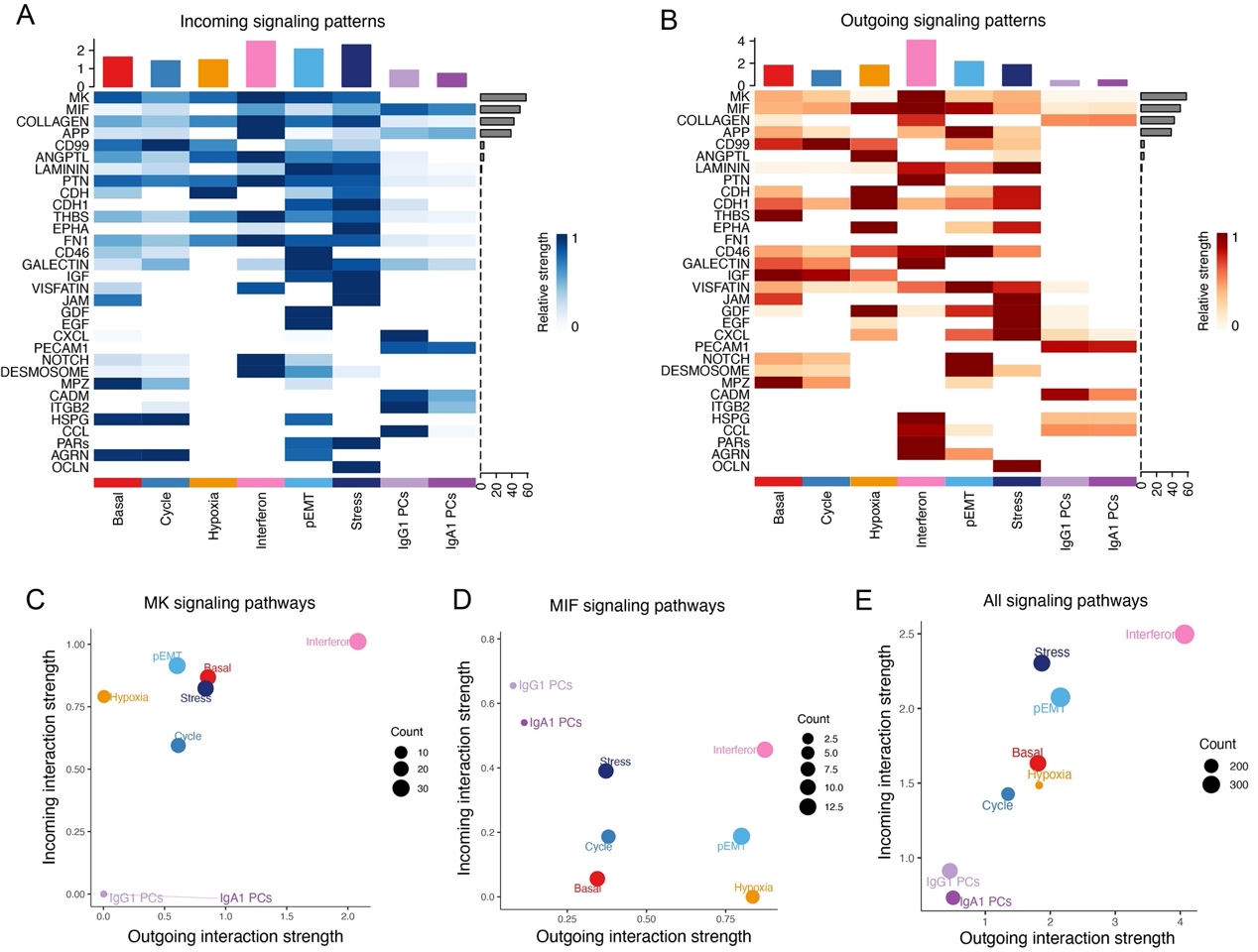
**

**FIGURE S8|** Signaling pathways enriched in plasma cell-tumor cell communication. **(A&B)** Heat map shows the signaling pathways enriched by each cell type in both incoming and outgoing signaling patterns. **(C-E)** Dot plots show the incoming and outgoing interaction strength of each cell type in MK/MIF signaling and all signaling pathways.


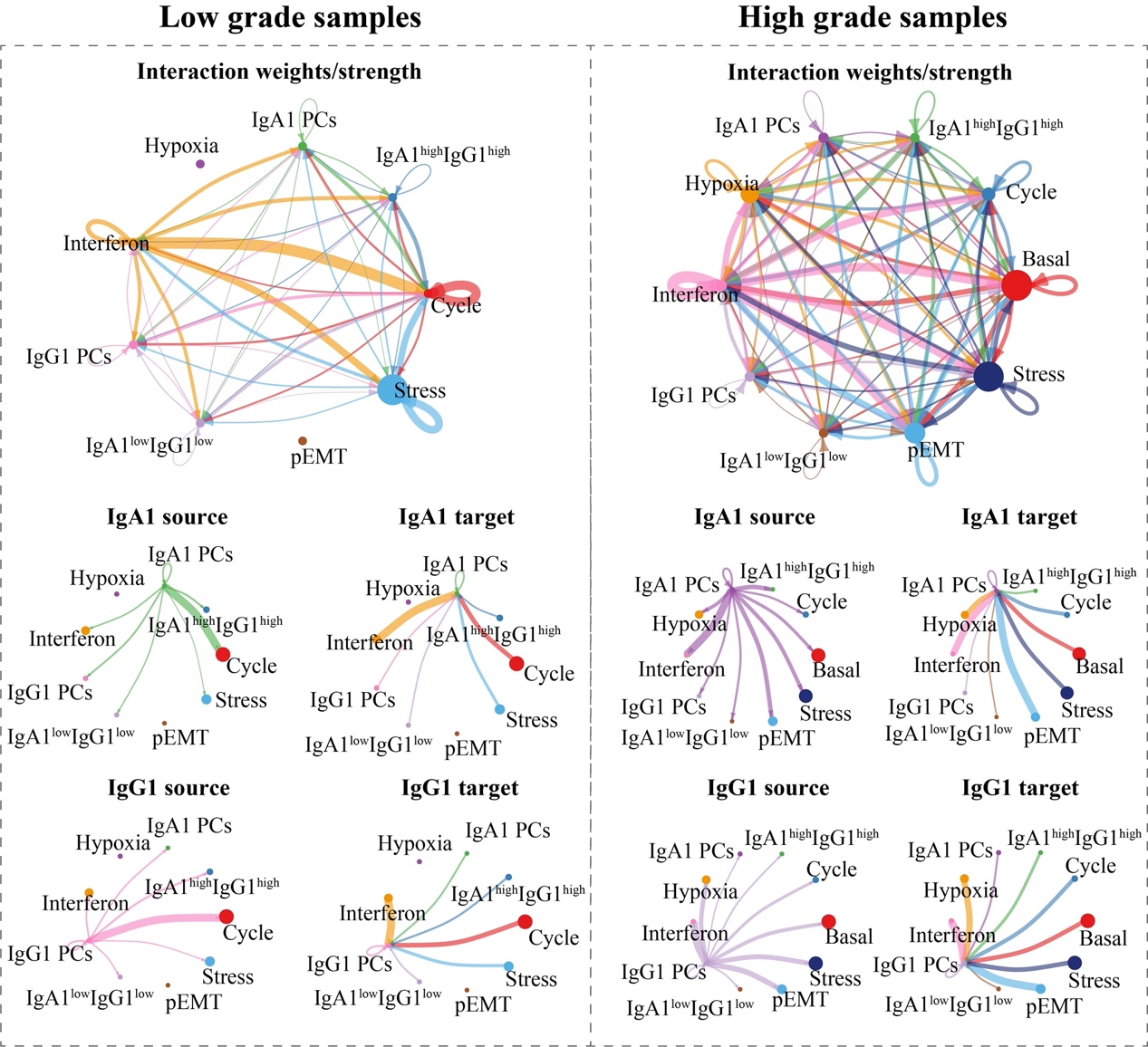


**FIGURE S9|** Comparison of PCs and tumor cell crosstalk in low- and high-grade bladder cancer samples

**
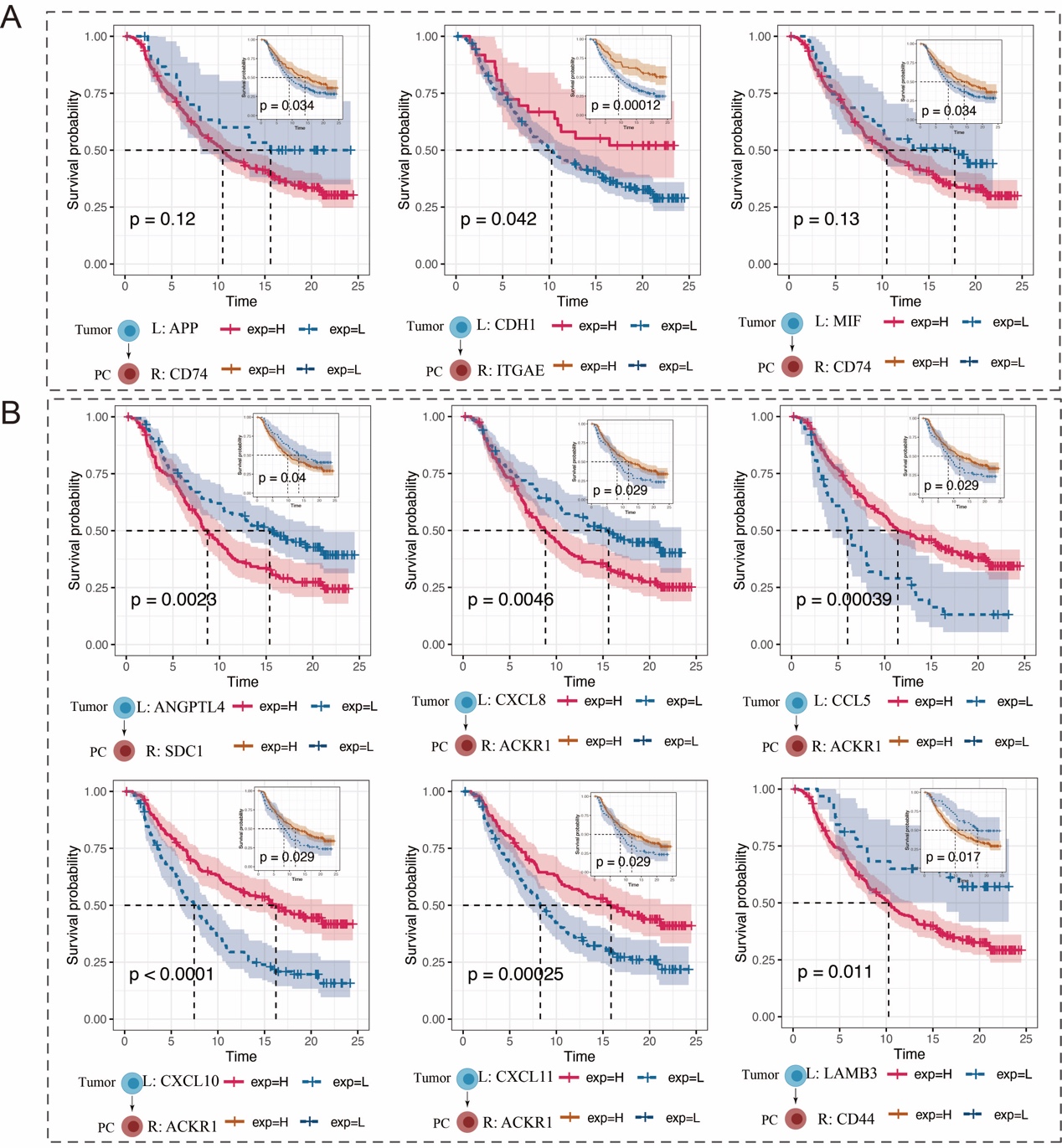
**

**FIGURE S10|** Survival analysis for ligand and receptor. **(A)** Overall survival analysis for selected L/R between tumor cells and PCs based on anti-PD-L1 treatment cohort IMvigor210. **(B)** Overall survival analysis for selected L/R between tumor cells and IgG1 PCs based on anti-PD-L1 treatment cohort IMvigor210.

**
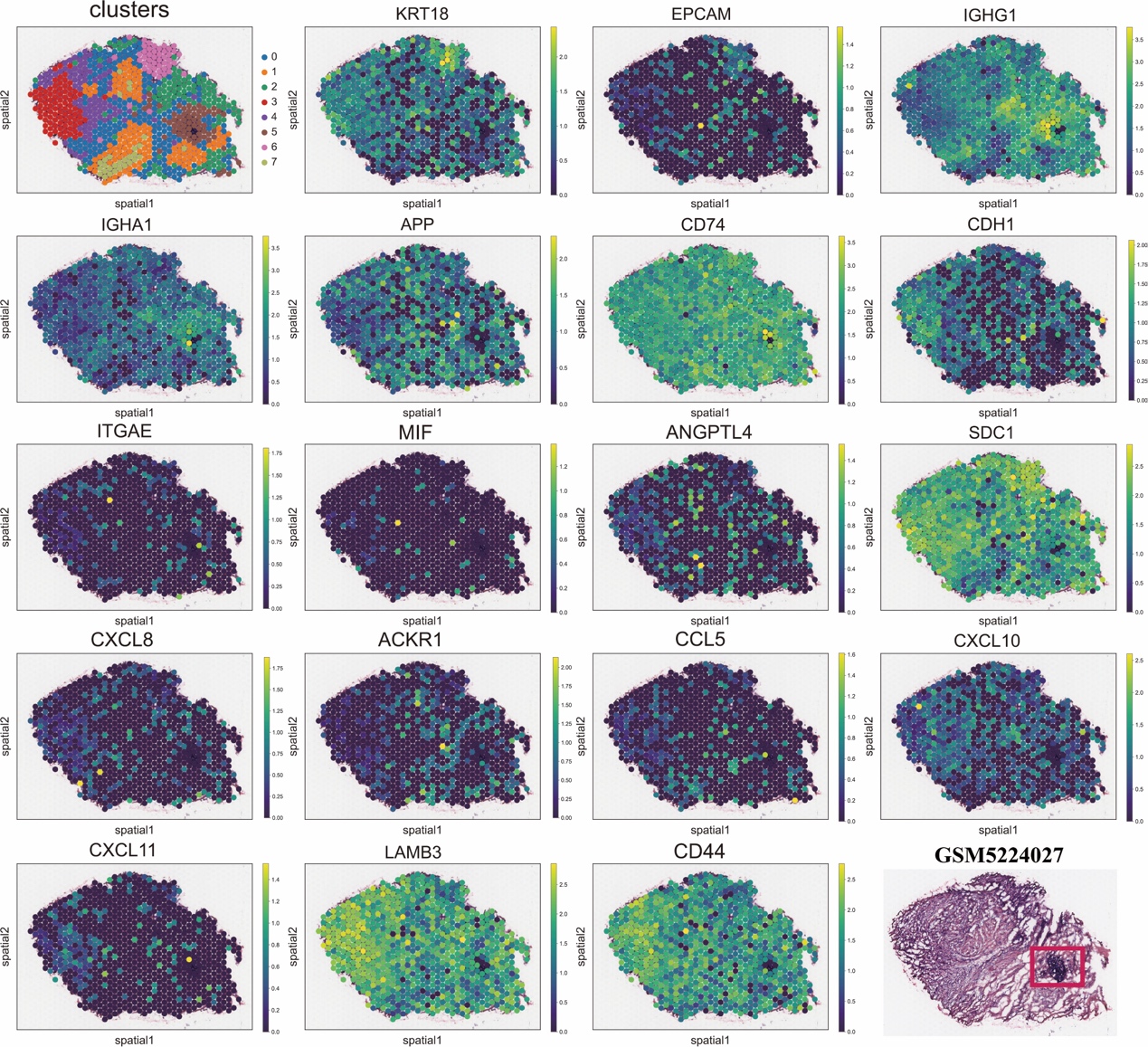
**

**FIGURE S11|** Spatial transcriptome cell clustering and expression assessment of selected genes based on sample GSM5224027.

**
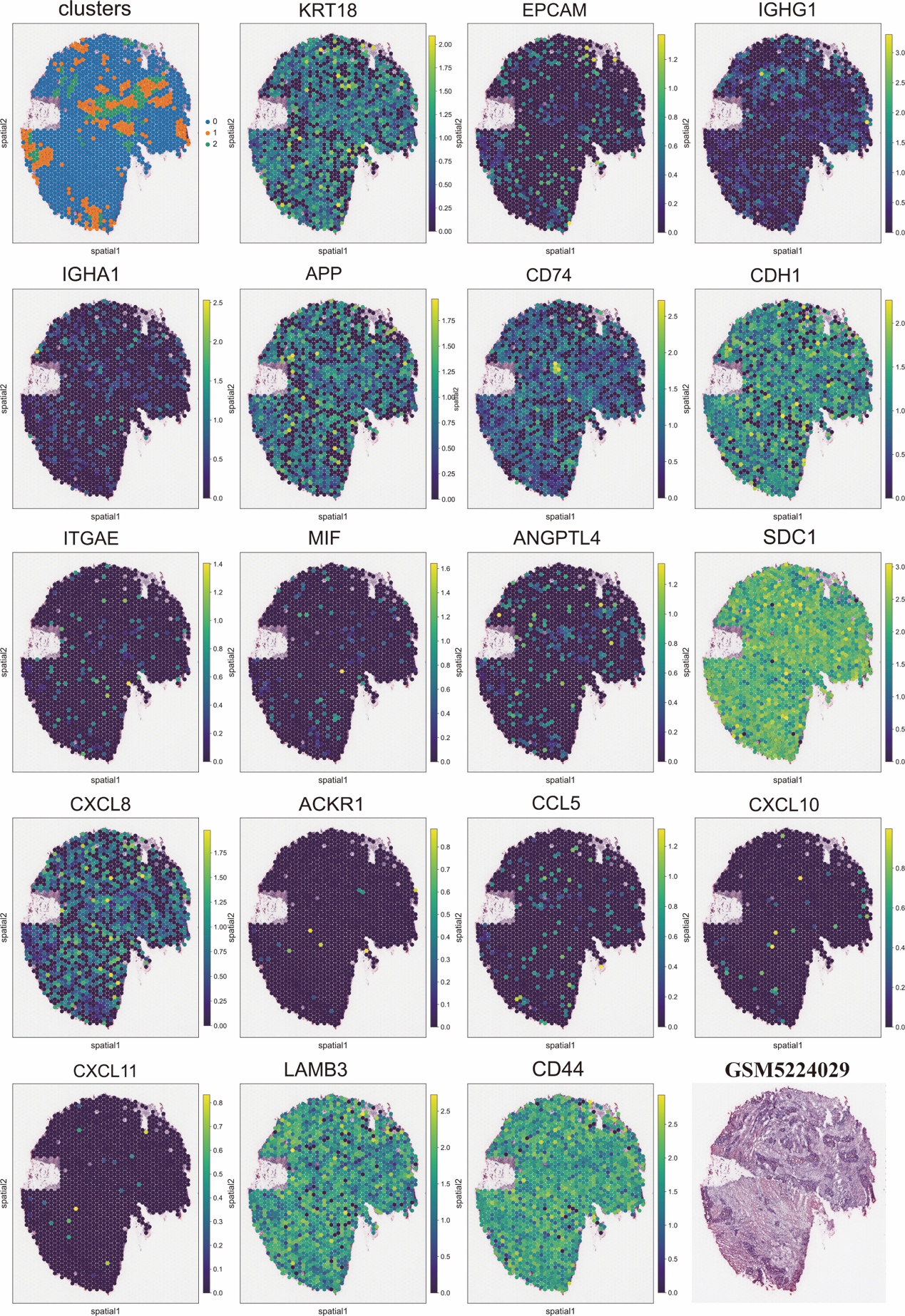
**

**FIGURE S12|** Spatial transcriptome cell clustering and expression assessment of selected genes based on validation sample GSM5224029.
